# Incidence of developmental disorders and special educational needs and disabilities in children in the UK

**DOI:** 10.1111/dmcn.16396

**Published:** 2025-07-16

**Authors:** Katherine Pettinger, Sarah Blower, Elaine Boyle, Catherine Hewitt, Lorna Fraser

**Affiliations:** ^1^ Department of Health Sciences University of York York UK; ^2^ Department of Population Health Sciences University of Leicester Leicester UK; ^3^ Cicely Saunders Institute of Palliative Care, Policy & Rehabilitation Kings College London London UK

## Abstract

**Aim:**

To investigate the incidence of developmental disorders (including cerebral palsy, attention‐deficit/hyperactivity disorder, and autism spectrum disorder) and special educational needs provision and to explore associations with gestational age and ethnicity.

**Method:**

Cumulative incidence of developmental disorders and special educational needs provision up to age 12 years/end of school year 7 respectively was explored using multivariable logistic regression in the Born in Bradford cohort, UK. Incidence rates of individual developmental disorders were calculated.

**Results:**

There were 13 172 children included in the analysis cohort. Birth before full term was associated with increased odds of developmental disorder compared with birth at full term: adjusted odds ratio (aOR) for those born before 34 weeks 2.22 (95% confidence interval [CI] 1.58–3.12); 34 to 36 weeks aOR 1.43 (95% CI 1.12–1.81); 37 to 38 weeks aOR 1.18 (95% CI 1.03–1.34). Effect sizes were larger among Pakistani heritage children: aOR for those born before 34 weeks 2.59 (95% CI 1.55–4.33); 34 to 36 weeks aOR 1.57 (95% CI 1.08–2.27); 37 to 38 weeks aOR 1.29 (95% CI 1.06–1.56). Unadjusted incidence rates of developmental disorders varied with ethnicity; compared with Pakistani heritage children, White British children had higher rates (per 1000 person‐years) of attention‐deficit/hyperactivity disorder (1.8, 95% CI 1.5–2.1 vs. 0.3, 95% CI 0.2–0.4), and lower incidences of learning disabilities (0.7, 95% CI 0.5–1.0 vs. 1.6, 95% CI 1.4–1.9).

**Interpretation:**

Irrespective of ethnicity, children born before full term are at increased risk of developmental disorders and/or special educational needs.

## Graphical abstract

The incidence of developmental disorders and special educational needs (SEN) provision were investigated, and associations with gestational age and ethnicity were explored, using the Born in Bradford cohort. Birth before full term was associated with increased odds of developmental disorder and/or SEN, which was even demonstrated in early term (37–38 weeks) born children, which persisted after adjustment for covariates, and in the analysis stratified by ethnicity. The incidence of individual developmental disorders varied with ethnicity.

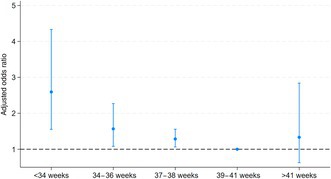

Plain language summary: https://onlinelibrary.wiley.com/doi/10.1111/dmcn.16444

**Abbreviations**
ASD: autism spectrum disorder
BiB: Born in Bradford
NICE: National Institute for Health and Care Excellence
SEN: special educational needs

**What this paper adds**

- Children born early term have increased likelihood of developmental disorders and special educational needs than those born full term.
- This relationship is also demonstrated among Pakistani heritage children.
- The relationships between covariates and special educational needs provision and developmental disorders are different.
- The incidence of individual developmental disorders varies with ethnicity.
- The incidence of developmental disorders overall is highest among White British children.

Children born before full term are at greater risk of developmental disorders than those born at full term^1^, ^2^ and are more likely than term‐born children to require special educational needs (SEN) provision in school.^3^ Delayed diagnoses of developmental disorders may lead to missed opportunities for early intervention, as well as causing distress to families.^4^, ^5^ Late instigation of educational support can compound impaired learning, resulting in disengagement from school.^6^ Anticipating the challenges children are likely to face could assist prompt identification of needs, facilitating improved health and well‐being.^5^

In England and Wales, the risk of preterm birth is markedly different between ethnicities, which is not fully explained by sociodemographic characteristics.^7^, ^8^ The risk is highest among Black females,^7^ although recently the largest proportional increase of preterm births was among Asian females.^8^ Despite this, the relation between preterm birth and developmental disorders among minoritized ethnic groups in the UK is unknown. Planning services requires an understanding of the local context and caution should be applied when extrapolating research internationally, owing to differences in health and education services, as well as population characteristics, terminology, and background rates of disorders.^9^ Most UK research exploring preterm birth and developmental outcomes is based on cohort studies where more than 80% of the participants are White.^10^, ^11^, ^12^

The UK Government defines SEN as a learning difficulty or disability which makes it harder for a child to learn compared with most children and young people of the same age.^3^ Since most research from the UK exploring effects of preterm birth uses educational attainment or SEN provision as the outcome measure,^10^, ^11^, ^12^ understanding the extent to which these overlap with developmental disorders will enable more effective counselling of parents and more efficient service planning.

This study reports the incidence of developmental disorders (as defined by the UK's National Institute for Health and Care Excellence [NICE]^13^) and SEN provision in a longitudinal birth cohort in Bradford, England: a large city with an ethnically diverse population. The aim was to describe the association between gestational age and the incidence of the full range of developmental disorders and SEN provision, then determine whether this varied between ethnicities.

## METHOD

### Data source

Born in Bradford (BiB) was established to investigate high levels of child mortality and morbidity.^14^ Between 2007 and 2010, 12 453 pregnant females were recruited to the cohort, which is broadly reflective of the city's maternal population; almost half the infants born in Bradford are of Pakistani descent.^15^ Most recruitment occurred during the oral glucose tolerance test, which is part of routine antenatal care in Bradford. Approximately 83% of pregnant females attended their test appointment, and more than 80% of these were recruited to the cohort study.^14^

Data collection for BiB is continuing and consists of linked routine health and education data and questionnaires for families. Ethical approval for BiB data collection was granted by Bradford Research Ethics Committee (reference 07/H1302/112).

### Participants

For inclusion in this analysis, children needed data from maternity records about gestational age and linked primary care data. Multiple births of higher order than twins were excluded. A subsample had linked education records (Figure S1). The date of data extract was 2nd February 2023 for primary care and 24th May 2023 for secondary care.

### Exposure variables

The key exposure was gestational age, in completed weeks.

### Outcome measures: developmental disorders

The developmental problems and disorders in the NICE guideline for developmental follow‐up of children born preterm^13^ were included: (1) cerebral palsy; (2) motor function problems; (3) learning disability; (4) special educational needs and low educational attainment (‘education problems’); (5) executive function problems; (6) speech, language, and communication problems; (7) hyperactivity, impulsivity, and inattention, including attention‐deficit/hyperactivity disorder; (8) autism spectrum disorder (ASD); (9) emotional and behavioural problems; (10) oromotor feeding problems; (11) visual impairment; (12) hearing impairment; (13) sleep apnoea; and (14) developmental delay.

The NICE guideline was chosen as a framework because it is a widely used evidence‐based document, recognized by a range of professionals involved in the care of children and young people, such as healthcare professionals and those working in education and social care. The guideline includes a broad range of conditions, reflecting the reality that developmental disorders often overlap and co‐occur.

In Bradford, while the initial point of contact about developmental concerns is likely to be the child's GP or health visitor, formal diagnoses of developmental disorders are usually made in the child development centre; these are then communicated back to the child's GP.

Clinical code lists corresponding to signs, symptoms, or diagnosis of developmental disorders were developed by the first author using published lists (see Tables S1 and S2) as well as by searching the Clinical Terminology browser and NHS Digital's Classifications browser.^16^ The participants' medical records were searched by the BiB data team to identify every occurrence of these codes. Codes relating to feeding problems recorded before 6 months of age were not included, as these were unlikely to represent oromotor feeding difficulties as described by NICE. The first code (by date) that appeared in either the primary or secondary care record was recorded as the incident code.

### Outcome measures: SEN provision

BiB receives data annually from all state‐funded schools in Bradford, including special schools (for children whose needs cannot be met in mainstream schools). For each academic year (from reception onwards), participants were classified as: (1) having an Education, Health, and Care Plan (previously a ‘statement’); (2) receiving SEN support (previously ‘School Action’ or ‘School Action Plus’); (3) no SEN.

There are four broad categories of SEN: (1) communicating and interacting; (2) cognition and learning; (3) social, emotional, and mental health difficulties; and (4) sensory and/or physical needs; but this detail was unavailable for the analysis. Children who require more than SEN support are eligible for an Education, Health, and Care Plan following a statutory assessment.^17^ The first recorded instance of a child being identified as requiring additional support due to SEN (Statement of SEN; an Education, Health, and Care Plan; School Action; School Action Plus; or SEN Support) was used to calculate cumulative incidence.

### Study covariates

Covariates included ethnicity, defined by BiB as White (English, Welsh, Scottish, Northern Irish ‘White British’; Irish; any other White background), Mixed (White and Black Caribbean; White and Black African; White and Asian; Any other Mixed background), Asian (Indian; Pakistani; Bangladeshi; Chinese; Any other Asian background), Black (African; Caribbean; Other Black, African, or Caribbean background), Any other ethnic group; sex (male/female); small for gestational age (birthweight <10th centile, yes/no); multiplicity (singleton/twin); socioeconomic position from a previous study (mutually exclusive groups based on latent class analysis using 19 variables including maternal education and receipt of means‐tested benefits measured during pregnancy)^18^ (least deprived and most educated; employed and not materially deprived; employed but no access to money; receiving benefits but coping; most deprived); maternal age at delivery (<21 years; 21–25 years; 26–30 years; 31–35 years; >35 years); maternal smoking during pregnancy (yes/no).

### Data derivation

To preserve anonymity, only the month and year of birth were provided. To calculate approximate age, days of birth were set to the first of each month. So that all the participants (except those who died or withdrew) had the same exposure time for cumulative incidence, a cut‐off point of the child's (approximated) 12th birthday was used for health records, and the end of academic year 7 (children aged 11–12 years) for education records.

### Statistical analysis

Summary statistics of gestational age by ethnicity were calculated.

Two different measures of disease frequency were calculated as follows. (1) The cumulative incidences of developmental disorders and SEN provision were calculated by dividing the number of new cases during the study period up to the 12th birthday (for developmental disorders) or end of year 7 (for SEN) by the total number of children at risk at the start of the study.^19^ (2) The incidence rate of individual developmental disorders, overall and stratified by ethnicity (Pakistani, White British), was calculated by dividing the numbers of children who had a code corresponding to a developmental disorder by the amount of at‐risk person‐time.^19^ Incidence rate was used as the measure of disease frequency when considering individual disorders, so as not to lose information. A child was only counted once as an incident case for each disorder^20^ but could be recorded as having multiple disorders (if applicable) for this analysis.

Multivariable logistic regression was used to investigate the relationship between gestational age (presented in five categories: <34 weeks [‘very preterm’ combined with ‘moderate preterm’]; 34–36 weeks [‘late preterm’]; 37–38 weeks [‘early term’]; 39–41weeks [‘full term’]; and>41weeks [‘post term’]) and the odds of developmental disorder and SEN provision up to the children's 12th birthdays or end of year 7 (developmental disorders and SEN respectively). Missing data within covariates were imputed using multiple imputation chained equations.^21^ Further information about imputation is provided in Tables S5 to S11. The analysis was then repeated stratified by the two largest ethnic groups (Pakistani, White British).

#### Sensitivity analyses

Some of the conditions included in the NICE guideline for the developmental follow‐up of children born preterm would not typically be classed as developmental disorders, and fall into the broader category of developmental problems, or conditions often associated with developmental disorders. A sensitivity analysis was performed, to determine whether the effects were similar when excluding sleep apnoea and only using diagnostic codes corresponding to DSM‐IV or ICD‐10 developmental disorders (i.e. excluding codes related to developmental ‘problems’, signs, or symptoms).

A secondary sensitivity analysis was undertaken to explore the robustness of the findings when accounting for loss to follow‐up (either due to death or withdrawal) using Kaplan–Meier estimation and Cox proportional hazards models including the same covariates outlined above.

All analyses were done using Stata/SE software version 18 (StataCorp, College Station, TX, USA).^22^

### Consent

Participants gave written informed consent to the research and to publication of the results.^15^

## RESULTS

There were 13 172 children in the analysis cohort (Figure S1), of whom 11 492 (87.2%) had linked education records and 11 368 SEN outcome data. Education data were unavailable if a child attended school out of area or attended private school. The analysis cohorts and the whole cohort had similar characteristics apart from multiplicity (Table 1). Within the analysis cohort there were 324 children who were withdrawn and 53 children who died; these data were included up until the point of death/withdrawal. The largest ethnic groups were Pakistani (*n* = 5788, 44.2%) and White British (*n* = 4624, 35.3%). The gestational age distribution varied between ethnic groups; 24.8% of Pakistani infants were born early term (37–38 weeks), compared with 19.2% of White infants (Table 2).

**TABLE 1:** Comparison of whole Born in Bradford cohort, analysis cohort, and subset with linked education records.

Characteristics	Whole cohort (*n* = 13 858)		Analysis cohort (*n* = 13 172)		Education subset of analysis cohort (*n* = 11 492)	
Gestational age	**Number**	**Percentage**	**Number**	**Percentage**	**Number**	**Percentage**
<28 weeks	32	0.2	21	0.2	17	0.2
28–31 weeks	118	0.9	99	0.8	77	0.7
32–33 weeks	110	0.8	94	0.7	78	0.7
34–36 weeks	646	4.8	606	4.6	527	4.6
37–38 weeks	3028	22.4	2950	22.4	2616	22.8
39–41 weeks	9407	69.5	9222	70.0	8026	69.8
>41 weeks	185	1.4	180	1.4	151	1.3
Missing	332		Excluded		Excluded	
Child's sex						
Male	6972	51.6	6801	51.6	5916	51.5
Female	6553	48.5	6371	48.4	5576	48.5
Missing	333		Nil		Nil	
Multiplicity						
Singletons	13 199	97.6	12 867	97.7	11 217	97.6
Twins	318	2.4	305	2.3	275	2.4
Higher order	9	0.1	Excluded		Excluded	
Missing	332		Nil		Nil	
SGA						
Not SGA	11 337	85.9	11 067	86.0	9621	85.8
SGA	1857	14.1	1795	14.0	1591	14.2
Missing	664		310		280	
Maternal age at infant's delivery, years:months						
Median (range), mean (SD)	27 (15–49), 27:6 (5:7)		27 (15–49), 27:6 (5:7)		27 (15–49), 27:6 (5:7)	
Missing	0		0		0	
Smoking during pregnancy						
Yes, any	1885	16.5	1782	16.4	1564	16.5
No	9568	83.5	9103	83.6	7916	83.5
Missing	2405		2287		2012	
Socioeconomic position						
Least deprived, most educated	2240	19.6	2096	19.3	1667	17.6
Employed not materially deprived	2273	19.9	2160	19.9	1863	19.7
Employed no access to money	1735	15.2	1656	15.3	1458	15.4
Benefits but coping	3347	29.4	3201	29.5	2902	30.7
Most deprived	1809	15.9	1730	16.0	1558	16.5
Missing	2454		2329		2044	
Child's ethnicity						
Pakistani	6001	43.6	5788	44.19	5299	46.13
White British	4889	35.6	4624	35.3	3915	34.08
Other	2857	20.8	2687	20.5	2272	19.8
Missing	111		73		6	

*Note:* Values are number (column percentage) unless stated otherwise. Children born <34 weeks were combined into one group for regression analyses owing to small numbers.

Abbreviations: SD, standard deviation; SGA, small for gestational age.

**TABLE 2:** Distribution of gestational age in analysis cohort by ethnicity.

Gestational age category	Pakistani	White British	Other	Total
<34 weeks	92	85	36	213
	1.6%	1.8%	1.3%	1.6%
34–36 weeks	249	221	136	606
	4.3%	4.8%	5.1%	4.6%
37–38 weeks	1,434	887	616	2,937
	24.8%	19.2%	22.9%	22.4%
39–41 weeks	3952	3350	1861	9163
	68.3%	72.5%	69.3%	70.0%
>41 weeks	61	81	38	180
	1.1%	1.8%	1.4%	1.4%
Total	5788	4624	2687	13 099
	100%	100%	100%	100%

### Cumulative incidence

There were 1497 out of 13 172 incident codes corresponding to developmental disorders and 3086 out of 11 368 ever received SEN provision giving the overall cumulative incidence of 11.4% (95% confidence interval [CI] CI 10.8–11.9%) and 27.1% (95% CI 26.3–28.0) respectively. Most (73.1%) children with a developmental disorder also received SEN provision. However, 59.5% of children who received SEN provision did not have a developmental disorder (Figure S2).

Cumulative incidence of developmental disorder and SEN provision varied with gestational age (highest incidence in the <34 weeks group), child's sex (higher incidence in males), multiplicity (slightly higher incidence in twins), small for gestational age (higher incidence if small for gestational age), socioeconomic position (highest incidence in most deprived group), ethnicity (highest incidence in White British group), and smoking (higher incidence if smoked) (Tables 3 and S14 Figure S3).

**TABLE 3:** Cumulative incidence of developmental disorder and SEN up to 12th birthday, and up to (and including) year 7 respectively and relationship with exposure and covariates.

	Developmental disorders			SEN provision		
	No developmental disorder	Developmental disorder	Total	Never any SEN provision	Ever any SEN provision	Total
**Gestational age category**						
<34 weeks	169	45	214	100	70	170
	79.0%	21.0%	100%	58.8%	41.2%	100%
34–36 weeks	517	89	606	330	189	519
	85.3%	14.7%	100%	63.6%	36.4%	100
37–38 weeks	2591	359	2950	1860	723	2583
	87.8%	12.2%	100%	72.01	28.0%	100%
39–41 weeks	8244	978	9222	5885	2062	7947
	89.4%	10.6%	100%	74.1%	26.0%	100%
>41 weeks	154	26	180	107	42	149
	85.6%	14.4%	100%	71.8%	28.2%	100%
Total	11 675	1497	13 172	8282	3086	11 368
	88.6%	11.4%	100%	72.9%	27.2%	100%
**Child's sex**						
Male	5796	1005	6801	3861	1997	5858
	85.2%	14.8%	100%	65.9%	34.1%	100%
Female	5879	492	6371	4421	1089	5510
	92.3%	7.7%	100%	80.2%	19.8%	100%
Total	11 675	1497	13 172	8282	3086	11 368
	88.6%	11.4%	100%	72.9%	27.2%	100%
**Multiplicity**						
Singleton	11 406	1461	12 867	8085	3010	11 095
	88.7%	11.4%	100%	72.9%	27.1%	100%
Twins	269	36	305	197	76	273
	88.2%	11.8%	100%	72.2%	27.8%	100%
Total	11 675	1497	13 172	8282	3086	11 368
	88.6%	11.4%	100%	72.9%	27.2%	100%
**SGA**						
Not SGA	9842	1225	11 067	7006	2505	9511
	88.9%	11.1%	100%	73.7%	26.3%	100%
SGA	1559	236	1795	1074	505	1579
	86.9%	13.2%	100%	68.0%	32.0%	100%
Total	11 401	1461	12 862	8080	3010	11 090
	88.6%	11.4%	100%	72.9%	27.1%	100%
**Mother's age at child's birth, years:months**						
Mean	27:6	27:6	27:6	27:8	27:1	27:6
SD	5:7	5:11	5:7	5:7	5:8	5:7
Median	27	27	27	27	27	27
Range	15–45	15–49	15–49	15–45	15–49	15–49
*n*	11 675	1497	13 172	8282	3086	11 368
**Socio‐economic position**						
Least deprived, most educated	1909	187	2096	1328	308	1636
	91.1%	8.9%	100%	81.2%	18.8%	100%
Employed, not deprived	1936	224	2160	1473	378	1851
	89.6%	10.4%	100%	79.6%	20.4%	100%
Employed, no access to money	1458	198	1656	1076	365	1441
	88.0%	12.0%	100%	74.7%	25.3%	100%
Benefits but coping	2800	401	3201	1963	910	2873
	87.4%	12.6%	100%	68.3%	31.7%	100%
Most deprived	1499	231	1730	979	567	1546
	86.5%	13.5%	100%	63.3%	36.7%	100%
Total	9602	1241	10 843	6819	2528	9347
	88.6%	11.5%	100%	73.0%	27.1%	100%
**Child's ethnicity**						
Pakistani	5158	630	5788	3806	1444	5250
	89.1%	10.9%	100%	72.5%	27.5%	100%
White British	4028	596	4624	2750	1126	3876
	87.1%	12.9%	100%	70.9%	29.1%	100%
Other	2416	271	2687	1722	516	2238
	89.9%	10.1%	100%	76.9%	23.1%	100%
Total	11 602	1497	13 099	8278	3086	11 364
	88.6%	11.4%	100%	72.8%	27.2%	100%
**Maternal smoking during pregnancy**						
No	8103	1000	9103	5816	2017	7833
	89.0%	11.0%	100%	74.3%	25.8%	100%
Yes	1536	246	1782	1034	512	1546
	86.2%	13.8%	100%	66.9%	33.1%	100%
Total	9636	1246	10 885	6850	2529	9379
	88.6%	11.5%	100%	73.0%	27.0%	100%

*Note:* See also Figure S3.

Abbreviations: SD, standard deviation; SEN, special educational needs; SGA, small for gestational age.

### Regression models

There were increased odds of developmental disorder and SEN provision in all groups born before full term, with relationships persisting after adjusting for covariates (Table 4). The odds ratio for developmental disorder and SEN provision was highest in the group born before 34 weeks: 2.22 (95% CI 1.58–3.12) and 2.10 (95% CI 1.52–2.90) respectively (Tables S3 and S4). The complete case analysis results can be seen in Tables S7 and S8 and were similar to these results using the imputed data sets.

**TABLE 4:** Stratified analyses, imputed data sets.

Developmental disorder									SEN							
	Pakistani heritage children				White British children				Pakistani heritage children				White British children			
	*n* = 5788				*n* = 4624				*n* = 5299				*n* = 3915			
	OR	95% CI		p	OR	95% CI		p	OR	95% CI		p	OR	95% CI		p
**Gestational age**																
<34 weeks	2.59	1.55	4.33	<0.001	1.34	0.75	2.39	0.3	2.87	1.78	4.62	<0.001	1.47	0.87	2.48	0.2
34–36 weeks	1.57	1.08	2.27	0.02	1.16	0.78	1.71	0.5	1.53	1.14	2.06	0.005	1.60	1.16	2.21	0.004
37–38 weeks	1.29	1.06	1.56	0.01	1.04	0.83	1.31	0.7	1.18	1.02	1.36	0.03	1.06	0.88	1.28	0.5
39–41 weeks	1 (reference)								1 (reference)							
>41 weeks	1.33	0.63	2.84	0.5	1.33	0.72	2.46	0.4	1.11	0.61	2.01	0.7	1.27	0.73	2.21	0.4
**Child's sex**																
Female	1 (reference)								1 (reference)							
Male	1.71	1.44	2.03	<0.001	2.47	2.05	2.99	<0.001	2.07	1.82	2.34	<0.001	2.39	2.06	2.77	<0.001
**SGA**																
Not SGA	1 (reference)								1 (reference)							
SGA	1.30	1.05	1.61	0.02	1.08	0.80	1.45	0.6	1.30	1.11	1.53	0.001	1.26	0.98	1.61	0.07
**Maternal age at delivery (years)**																
<21	1.01	0.67	1.52	0.95	0.92	0.66	1.27	0.6	1.27	0.95	1.70	0.1	1.12	0.86	1.45	0.4
21–25	1.01	0.79	1.29	0.94	1.12	0.85	1.49	0.4	1.06	0.89	1.27	0.5	1.07	0.85	1.35	0.6
26–30	1.06	0.84	1.34	0.6	1.01	0.76	1.35	0.9	1.06	0.89	1.26	0.5	1.00	0.80	1.27	0.95
31–35	1 (reference)								1 (reference)							
>35	1.33	0.97	1.83	0.08	1.40	1.00	1.97	0.05	0.96	0.74	1.23	0.7	1.31	0.98	1.75	0.07
**Socio‐economic position**																
Most educated, least deprived	1 (reference)								1 (reference)							
Employed not materially deprived	1.14	0.80	1.64	0.5	1.22	0.88	1.70	0.2	0.97	0.72	1.32	0.9	1.13	0.87	1.48	0.4
Employed no access to money	1.07	0.78	1.47	0.7	1.62	1.13	2.33	0.009	1.27	0.98	1.65	0.08	1.60	1.18	2.17	0.002
Benefits but coping	1.16	0.90	1.50	0.3	2.05	1.40	3.00	<0.001	1.77	1.43	2.18	<0.001	2.56	1.89	3.47	<0.001
Most deprived	1.03	0.74	1.43	0.9	2.26	1.59	3.22	<0.001	2.00	1.55	2.58	<0.001	3.12	2.33	4.18	<0.001
**Maternal smoking in pregnancy**																
Non‐smoking	1 (reference)								1 (reference)							
Smoking (any)	1.11	0.70	1.76	0.5	1.05	0.85	1.30	0.6	0.98	0.65	1.48	0.9	1.06	0.88	1.27	0.5

*Note:* Results are based on logistic regression models with the two outcomes (developmental disorder, SEN) occurring at any point during the follow‐up period and the noted predictor variables. Imputation details: Developmental disorder. Pakistani heritage children: imputations, 20; average RVI, 0.05; largest FMI, 0.17. See also Figure 1. White British children: imputations, 16; average RVI, 0.05; largest FMI, 0.15.

SEN provision. Pakistani heritage children: imputations, 20; RVI, 0.09; largest FMI, 0.30. See also Figure 4. White British children, imputations, 17, average RVI, 0.05, largest FMI, 0.18.

Abbreviations: CI, confidence interval; FMI, fraction of missing information; OR, odds ratio; RVI, relative variance increased; SEN, special educational needs; SGA, small for gestational age.

**FIGURE 1:**
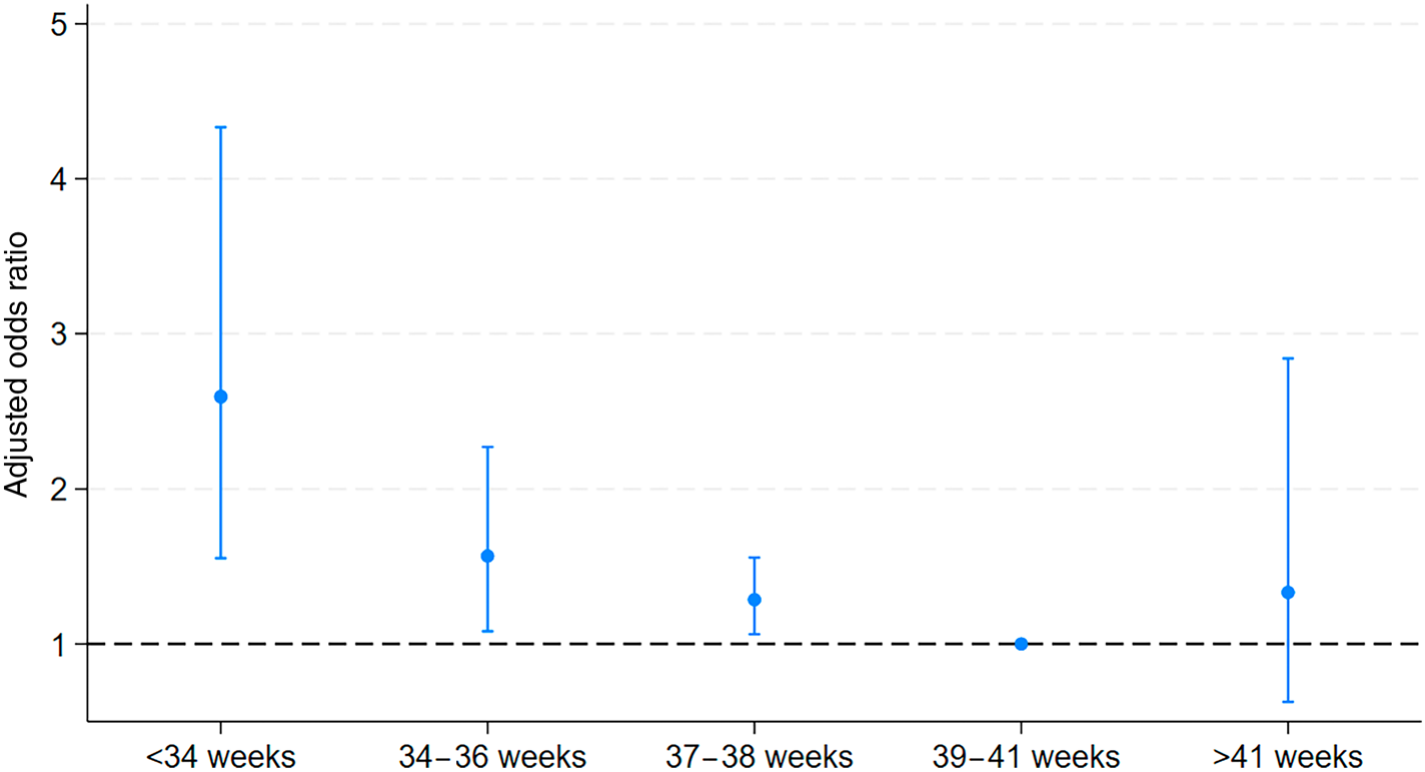
Stratified analysis of Pakistani heritage children. Coefficient plot showing adjusted odds ratios and 95% confidence intervals of developmental disorder by gestational age.

The relationship between gestational age and odds of developmental disorder and SEN provision were similar in the stratified analysis compared with the main analysis. All effect sizes stayed the same or were larger and remained statistically significant in the analysis of Pakistani heritage children (Table 4 and Figure 1). In the analysis of White British children, none of the odds ratios remained statistically significant, except for SEN provision in the 34‐week to 36‐week group (Table 4).

### Sensitivity analysis

While the cumulative incidence was lower using the restricted case ascertainment strategy, the effect sizes were only marginally different (see Table S15).

The results of the Kaplan–Meier estimation and Cox regression are presented in Tables S16 to S21 and Figures S4 to S7; the hazard ratios show similar trends to the odds ratios from the logistic regression models.

### Unadjusted incidence rate of individual developmental disorders

There were a total of 1640 incident (i.e. first) codes within the health records, with 164 823.2 person‐years of time at risk. The overall incidence rate of developmental disorders was 10.0 (95% CI 9.5–10.4) per 1000 person‐years. The highest incidence rate was for speech, language, and communication disorders with ASD next, and cerebral palsy the lowest (Table S12). There were no clinical codes relating to executive function problems.

**FIGURE 2:**
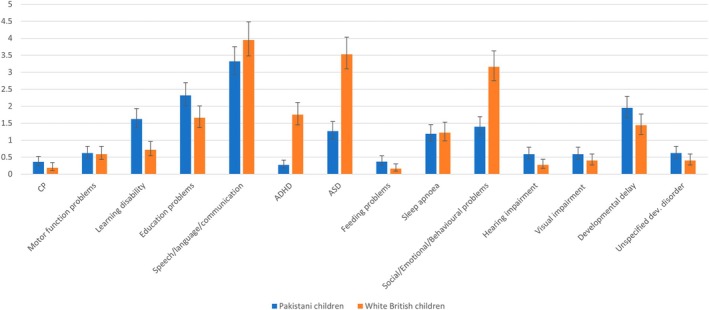
Bar chart showing incidence rate of each developmental disorder, per 1000 person‐years, by ethnicity. Abbreviations: ADHD, attention‐deficit/hyperactivity disorder; ASD, autism spectrum disorder; CP, cerebral palsy; dev., developmental.

White British children had a higher incidence rate (per 1000 person‐years) of ASD (3.5, 95% CI 3.1–4.0 vs. 1.3, 95% CI 1.0–1.6), attention‐deficit/hyperactivity disorder (1.8, 95% CI 1.5–2.1 vs. 0.3, 95% CI 0.2–0.4), and social, emotional, and behavioural problems (3.2 95% CI 2.8–3.6 vs. 1.4 95% CI 1.2–1.7) than Pakistani heritage children respectively. Pakistani heritage children had a higher incidence of learning disabilities (1.6, 95% CI 1.4–1.9 vs. 0.7, 95% CI 0.5–1.0) and hearing impairments (0.59, 95% CI 0.45–0.79 vs. 0.27, 95% CI 0.17–0.44) than White British children (Figure 2 and Table S13).

## DISCUSSION

This is the largest UK study exploring the association between gestational age and all developmental disorders. Stratified analysis showed that, regardless of ethnicity, children born before full term are at risk of developmental disorders, with larger effect sizes demonstrated among Pakistani heritage children. Similar relationships between gestational age and SEN provision were demonstrated, but most children who received SEN provision did not also have a developmental disorder. The incidence of developmental disorders was lower among Pakistani heritage children than White British children, largely driven by lower incidence rates of ASD and social, emotional, and behavioural problems.

This study confirms the risk of developmental disorders extends to children born late preterm and early term, which persists in the analysis stratified by ethnicity. Given ‘preterm pathologies’ such as intraventricular haemorrhage and retinopathy of prematurity are rare in these children, it had been suggested this relationship could reflect antenatal problems (e.g. pre‐eclampsia), which predispose to both preterm birth and developmental disorder;^23^ or social deprivation, because poverty is associated with both preterm birth and impaired brain growth.^24^ However, multivariable modelling, adjusting for small for gestational age (an indicator of antenatal pathology) and deprivation, suggests a true effect of immaturity, possibly because of disruption of healthy neural connections and brain maturation.^2^, ^23^

While these findings seem consistent with recent literature,^1^, ^2^ direct quantitative comparison with other sources is challenging. Mitha et al. found the incidence rate of neurodevelopmental impairment ranged from 4.6 per 1000 person‐years among the group born at full term, to 9.2 per 1000 person‐years in the moderate preterm group.^1^ However, this was a Swedish cohort, from a different epoch (born 1998–2012), with a different case ascertainment strategy, not including all developmental disorders. Despite this, it is plausible that Bradford's incidence of developmental disorder truly is higher, given known associations between poverty and developmental disorders.^24^ The cumulative incidence of SEN up to year 7 was comparable to other large studies using data from England; Libuy et al. found 29.5% of children born at 40 weeks received SEN provision at some point between reception and year 6 compared with 38.1% of children born at 36 weeks and 45.9% of children born at 32 weeks.^11^

The proportion of infants born before full term varied with ethnicity; a larger proportion of White British infants (72.5%) were born at full term compared with Pakistani infants (68.3%). Early term birth is common (24.8%) among Pakistani females (19.2% among White British females), but in the stratified analysis early term‐born Pakistani heritage children had an odds ratio for developmental disorder of 1.29 (1.06–1.56); while early term delivery may be more common among minoritized ethnic groups, it should not be seen as ‘harmless’.

In contrast to existing literature,^2^, ^15^ the overall incidence of developmental disorder was higher among White British than Pakistani heritage children, largely related to the higher incidence rate of ASD, attention‐deficit/hyperactivity disorder, and social, emotional, and behavioural problems seen in White British children, perhaps related to cultural norms, clinician biases, or barriers to service access and provision.^25^, ^26^ There could be a genetic component because ASD and attention‐deficit/hyperactivity disorder have a heritability of 50% and 74% respectively.^27^, ^28^ However, this seems unlikely because among the least deprived groups the cumulative incidence of developmental overall is very similar between Pakistani and White British children (Figure S3), suggesting underdiagnosis of some conditions among some deprived communities. Compared with White British children, Pakistani heritage children had a higher incidence of rarer conditions such as learning disability, which can profoundly impact a child's life; people with learning disabilities often suffer from significant mental and physical health issues and have reduced life expectancy.^29^

The bulk of UK research on the effects of preterm birth either uses educational achievement or SEN provision as its outcome measure,^10^, ^11^ or focuses on a single diagnosis, for example ASD.^12^ This is the first UK study to use both education data and a full range of developmental disorders to measure outcomes among children born before full term. It demonstrates that most (73.1%) children with a developmental disorder in their medical records also received SEN provision, but many (59.5%) children who received SEN provision did not also have a developmental disorder. In 2023, ‘social, emotional, and mental health difficulties’ were the second most common type of SEN, after speech, language, and communication difficulties; mental health difficulties were not one of this study's outcome measures.^17^ There are many non‐health‐related factors that have been shown to affect requirement for SEN provision, such as the child's age within their academic year group (with SEN provision more likely if summer‐born), maternal education level, eligibility for free school meals, and whether English is an additional language for the child.^30^ Children who require SEN provision are more likely to come from deprived homes; deprivation may be seen as both a cause as well as a consequence of SEN.^31^ Indeed, univariable analyses showed that the effect of deprivation was more pronounced for SEN compared with developmental disorders (Figure S3). The adjusted models investigating gestational age and developmental disorder, and gestational age and SEN provision, are not identical. The effect of socioeconomic position was more pronounced in the SEN provision model, and the effect of maternal age was different: the highest risk group in the developmental disorder model was those older than 35 years whereas in the SEN provision model it was those younger than 21 years, reflecting the differences underlying the labels of ‘SEN’ and ‘developmental disorder’; research relating to the association between gestational age and SEN provision cannot be automatically extrapolated to developmental disorders.

This study had limitations. It relied upon the comprehensiveness and accuracy of the coding within participants' medical records; if a child did not attend either primary care or secondary care and did not subsequently have a code related to a developmental disorder entered in their record, they would not be recorded as a case. The case ascertainment strategy was broad, to account for unusual coding practices and differences in how ethnic groups access health care.^32^ Some conditions defined here as ‘developmental disorders’ do not fulfil stricter definitions of developmental disorder; this was explored in the sensitivity analysis. Although the dose–response relationship between gestational age and the outcomes makes causality likely, as with all observational studies this cannot be certain.

Within the main analysis cohort of 13 172 children, 324 were withdrawn from the study and 53 died. The cumulative incidence calculations and logistic regression models do not account for these censored cases, which could lead to an inaccurate estimation of cumulative incidence, or misleading odds ratios from the logistic regression. The estimates derived from Kaplan–Meier analysis were consistently higher than those reported using crude proportions (Table 3). This is to be expected, since the cumulative incidence calculations included all participants in the denominator, potentially underestimating the true probability of developmental disorders or SEN. However, given the relatively small number of censored observations and the similarity of results with the Cox regression analysis, the overall findings remain robust.

Birth before full term is associated with some syndromes and congenital anomalies such as Down syndrome and spina bifida, which are also known to result in developmental disorders.^33^ This was not accounted for in the analyses.

The BiB cohort is largely bi‐ethnic. Apart from the Pakistani heritage and White British groups, the remaining ethnic groups were too small to explore. In the stratified analysis, the confidence intervals for the effect sizes in the analysis of White British children expanded to include the null value of 1, probably because of small numbers since the direction and size of effect remained similar to the main model.

There were very few children born extremely preterm, partly because of the cohort recruitment strategy (at 26 weeks).^15^ However, outcomes of extreme preterm birth were not the main focus of this paper; they have different pathophysiological processes and have been investigated elsewhere.

There are several implications resulting from this study. Birth before full term is more common in minoritized ethnic groups but remains associated with increased risk of both developmental disorder and SEN provision. Early term delivery should not be normalized; it should be avoided unless there is a compelling indication.

Children born late preterm do not routinely receive developmental follow‐up; guidelines recommend parents should contact their GP or health visitor if they are concerned.^15^ Parents' ability or desire to access advice may be influenced by their previous experiences of health care and their social, cultural, or ethnic background,^34^ exacerbating inequalities. An expansion of routine follow‐up would need to be balanced against cost and time implications. A pragmatic approach might be to target children with additive risk factors, or to use screening questionnaires such as the Parent Report of Children's Abilities‐Revised. Education professionals aware of the difficulties associated with preterm birth may be able to identify problems. While better integration of health and education services is desirable, once a child starts formal education the opportunity for truly ‘early’ intervention may have passed. Health visitors (specialist community public health nurses) would be ideally placed to act on concerns about children's development; however, the service is already overstretched.^35^

Overall, the developmental disorders with the highest incidence rates were: (1) speech, language, and communication disorders; (2) ASD; and (3) social, emotional, and behavioural problems; however, this varied with ethnicity. Understanding local context is important when planning services, to meet the needs of the community.

Parents should be empowered with information about the risks facing their children, but it is not clear when or how this information should be delivered. Qualitative research with families who have lived experience of the diagnostic journey would be invaluable.

## CONCLUSION

This study shows a clear association between gestational age and developmental disorders and SEN provision, even in the early term group, irrespective of ethnicity, which persisted after adjustment. The incidence rate of individual developmental disorders varied between Pakistani heritage and White British children. Knowing who is at risk of which developmental disorders enables effective service planning and early intervention.

## FUNDING INFORMATION

Born in Bradford received funding from a joint grant from the UK Medical Research Council (MRC) and UK Economic and Social Science Research Council (ESRC) (MR/N024391/1); the British Heart Foundation (CS/16/4/32482); a Wellcome Infrastructure Grant (WT101597MA); the National Institute for Health Research under its Applied Research Collaboration for Yorkshire and Humber (NIHR200166). The National Institute for Health Research Clinical Research Network provided research delivery support for this study. The views expressed in this publication are those of the authors and not necessarily those of the National Institute for Health Research or the Department of Health and Social Care. Dr. Pettinger, Doctoral Research Fellow, is funded by the National Institute for Health and Social Care Research (NIHR) for this research (award reference NIHR301738). The funder has no role in the interpretation of data, writing of the report, or decision to submit for publication. The views expressed in this publication are those of the authors and not necessarily those of the NIHR, NHS, or the UK Department of Health and Social Care.

## CONFLICT OF INTEREST STATEMENT

The authors have stated that they had no interests that might be perceived as posing a conflict or bias.

## Supporting information


**Figure S1:** Sample selection flow chart. NB – several females had more than one 'BiB' pregnancy and birth.

**Figure S2:** Venn diagram to show the proportions of children with a developmental disorder in their primary care, secondary care records, or any Special Educational Need (SEN) in their educational records, and how these intersect. *N* = 11,368.

**Figure S3:** Cumulative incidence of developmental disorder up to age 12 (left side) and Special Educational Needs (SEN) up to (and including) year 7 (right side) according to gestational age and covariates. Green dashed lines indicate 95% confidence intervals (CIs) for top four graphs; in the bottom six graphs the dot shows estimate and vertical lines show 95% CIs.

**Figure S4:** Time (age; 0 = birth) to developmental disorder by gestational age group.

**Figure S5:** ime (age; 0 = birth) to developmental disorder by gestational age group, with post term group removed for readability.

**Figure S6:** Time (age) to Special Educational Need by gestational age group.

**Figure S7:** Time (age) to Special Educational Need by gestational age group, with post term group removed for readability.

**Data S1:** **Table S1:** ICD‐10 codes and terms.
**Table S2:** Clinical terms version 3 (CTV3) ‘Read’ codes and terms.
**Table S3:** Results of logistic regression model including all co‐variables of interest, imputed dataset, odds ratio of developmental disorder by gestational age.
**Table S4:** Results of logistic regression model including all co‐variables of interest, imputed dataset, odds ratio of special educational need provision by gestational age.
**Table S5:** Proportion of missing data.
**Table S6:** Multiple imputation chained equations diagnostics for main analysis.
**Table S7:** Results of logistic regression model including all co‐variables of interest, odds ratio of developmental disorder.
**Table S8:** Results of logistic regression model including all co‐variables of interest, odds ratio of special educational need provision by gestational age.
**Table S9:** Results of logistic regression model for developmental disorder stratified by two largest ethnic groups – Complete case analysis.
**Table S10:** Results of logistic regression model for special educational needs provision stratified by two largest ethnic groups – Complete case analysis.
**Table S11:** Multiple imputation chained equations diagnostics for analysis stratified by ethnicity.
**Table S12:** Incidence rate of each disorder/group of disorders, sorted by incidence rate.
**Table S13:** Incidence rate per 1000 person‐years of developmental disorders by ethnicity.
**Table S14:** Cumulative incidence of developmental disorder using unrestricted and restricted case ascertainment strategies.
**Table S15:** Odds ratio of developmental disorder according to gestational age.
**Table S16:** Kaplan–Meier estimates of time to first developmental disorder diagnosis by gestational age group.
**Table S17:** Kaplan–Meier estimates of time to first special educational needs by gestational age group.
**Table S18:** Cox proportional hazards model for time to first diagnosis of developmental disorder (complete case analysis).
**Table S19:** Cox proportional hazards model for time to first diagnosis of developmental disorder including time‐varying covariates using an interaction with log(time) (complete case analysis).
**Table S20:** Cox proportional hazards model for time to first special educational needs (complete case).
**Table S21:** Cox proportional hazards model for time to special educational needs including time‐varying covariates using an interaction with log(time).

## Data Availability

Data are not available from the authors but can be accessed via Born in Bradford as described online: https://borninbradford.nhs.uk/research/how‐to‐access‐data/.
