# Supplementary material for: Incidence of developmental disorders and special educational needs and disabilities in children in the UK
Source: Dev Med Child Neurol. 2025 Jul 16;68(2):263–75. doi: 10.1111/dmcn.16396 (PMC12766549; doi:10.1111/dmcn.16396)
Supplement: Supplementary file 1 — Figure S1: Sample selection flow chart. NB – several females had more than one 'BiB' pregnancy and birth. [file DMCN-68-263-s004.docx]

# Supporting Information

Mothers recruited to Born in Bradford during pregnancy = 12,453

Born in Bradford pregnancies = 13,361

**Excluded:**

Multiple births higher order than twins (n = 9)

Missing gestational age (n=332)

No primary care records (n = 345)

Births in cohort = 13,858

(from linking file)

Analysis cohort n = 13,172

Subset with linked education records n = 11,492

Figure S1 – Sample selection flow chart. NB – several women had more than one “BiB” pregnancy and birth.
