# Supplementary material for: Incidence of developmental disorders and special educational needs and disabilities in children in the UK
Source: Dev Med Child Neurol. 2025 Jul 16;68(2):263–75. doi: 10.1111/dmcn.16396 (PMC12766549; doi:10.1111/dmcn.16396)
Supplement: Supplementary file 2 — Figure S2: Venn diagram to show the proportions of children with a developmental disorder in their primary care, secondary care records, or any Special Educational Need (SEN) in their educational records, and how these intersect. N = 11,368. [file DMCN-68-263-s001.docx]

*Figure S2 – Venn diagram to show the proportions of children with a developmental disorder in their primary care, secondary care records, or any Special Educational Need (SEN) in their educational records, and how these intersect. N = 11,368*


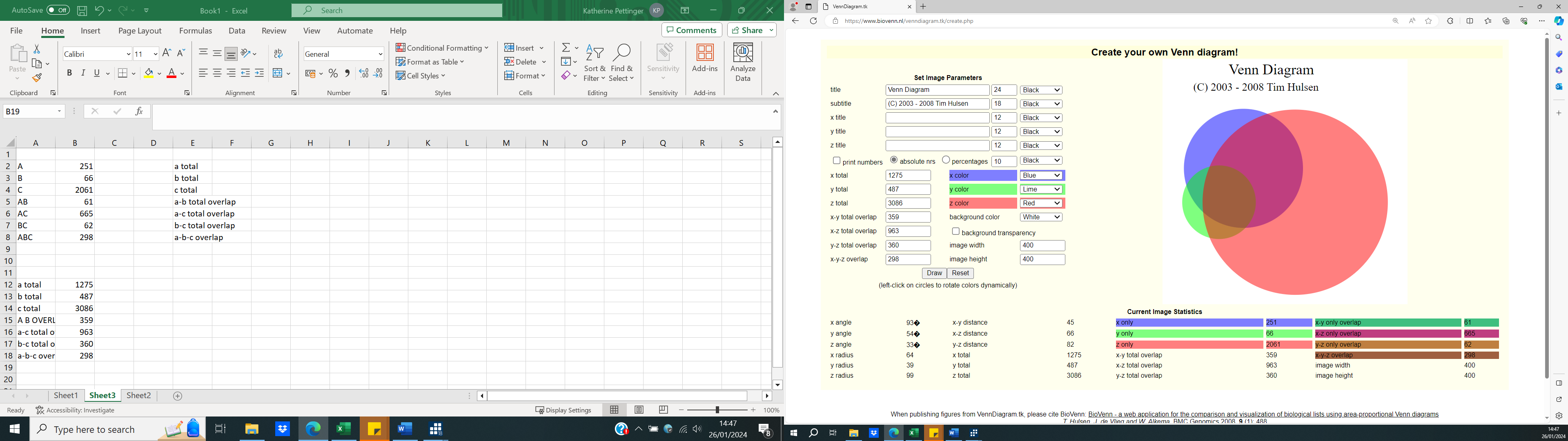


251 (2.2%)

Primary healthcare code

Secondary healthcare code

Primary & secondary healthcare codes & SEN

298 (2.6%)

Primary & secondary healthcare codes

61 (0.5%)

Secondary healthcare code & SEN

62 (0.5%)

66 (0.6%)

Primary healthcare code & SEN

665 (5.8%)

SEN

2,061 (18.1%)
