# Supplementary material for: Incidence of developmental disorders and special educational needs and disabilities in children in the UK
Source: Dev Med Child Neurol. 2025 Jul 16;68(2):263–75. doi: 10.1111/dmcn.16396 (PMC12766549; doi:10.1111/dmcn.16396)
Supplement: Supplementary file 3 — Figure S3: Cumulative incidence of developmental disorder up to age 12 (left side) and Special Educational Needs (SEN) up to (and including) year 7 (right side) according to gestational age and covariates. Green dashed lines indicate 95% confidence intervals (CIs) for top four graphs; in the bottom six graphs the dot shows estimate and vertical lines show 95% CIs. [file DMCN-68-263-s002.docx]

*Figure S3 - Cumulative incidence of developmental disorder up to age 12 (left side) and Special Educational Needs (SEN) up to (and including) Year 7 (right side) according to gestational age and covariates. Green dashed lines indicate 95% confidence intervals (CIs) for top four graphs; in the bottom 6 graphs the dot shows estimate and vertical lines show 95% CIs.*

*
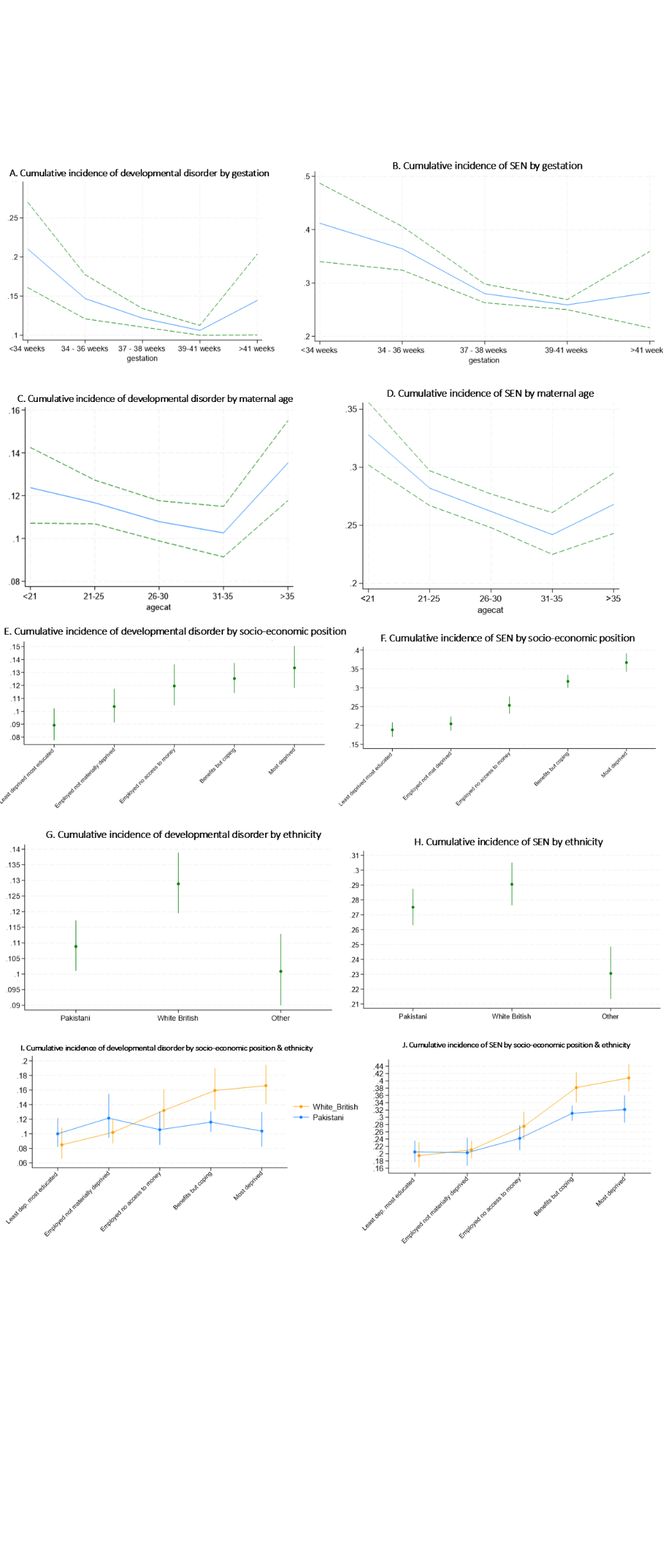
*
