# Supplementary material for: Incidence of developmental disorders and special educational needs and disabilities in children in the UK
Source: Dev Med Child Neurol. 2025 Jul 16;68(2):263–75. doi: 10.1111/dmcn.16396 (PMC12766549; doi:10.1111/dmcn.16396)
Supplement: Supplementary file 4 — Figure S4: Time (age; 0 = birth) to developmental disorder by gestational age group. [file DMCN-68-263-s006.docx]

*Figure S4 – Time (age; 0 = birth) to developmental disorder by gestational age group*
