# Supplementary figures and images for: Incidence of developmental disorders and special educational needs and disabilities in children in the UK
Source: Dev Med Child Neurol. 2025 Jul 16;68(2):263–75. doi: 10.1111/dmcn.16396 (PMC12766549; doi:10.1111/dmcn.16396)

*Figure S6 – Time (age) to Special Educational Need by gestational age group*


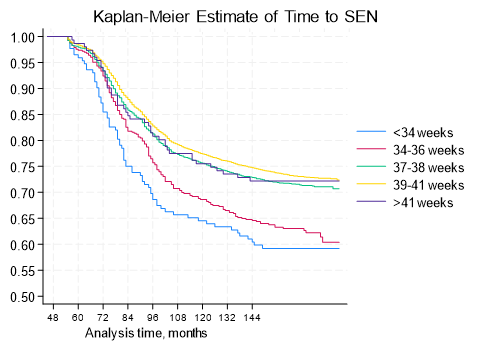

Supplement: Supplementary file 6 — Figure S6: Time (age) to Special Educational Need by gestational age group. [file DMCN-68-263-s005.docx]
