# Supplementary material for: Incidence of developmental disorders and special educational needs and disabilities in children in the UK
Source: Dev Med Child Neurol. 2025 Jul 16;68(2):263–75. doi: 10.1111/dmcn.16396 (PMC12766549; doi:10.1111/dmcn.16396)
Supplement: Supplementary file 8 — Data S1: Table S1: ICD‐10 codes and terms. Table S2: Clinical terms version 3 (CTV3) ‘Read’ codes and terms. Table S3: Results of logistic regression model including all co‐variables of interest, imputed dataset, odds ratio of developmental disorder by gestational age. Table S4: Results of logistic regression model including all co‐variables of interest, imputed dataset, odds ratio of special educational need provision by gestational age. Table S5: Proportion of missing data. Table S6: Multiple imputation chained equations diagnostics for main analysis. Table S7: Results of logistic regression model including all co‐variables of interest, odds ratio of developmental disorder. Table S8: Results of logistic regression model including all co‐variables of interest, odds ratio of special educational need provision by gestational age. Table S9: Results of logistic regression model for developmental disorder stratified by two largest ethnic groups – Complete case analysis. Table S10: Results of logistic regression model for special educational needs provision stratified by two largest ethnic groups – Complete case analysis. Table S11: Multiple imputation chained equations diagnostics for analysis stratified by ethnicity. Table S12: Incidence rate of each disorder/group of disorders, sorted by incidence rate. Table S13: Incidence rate per 1000 person‐years of developmental disorders by ethnicity. Table S14: Cumulative incidence of developmental disorder using unrestricted and restricted case ascertainment strategies. Table S15: Odds ratio of developmental disorder according to gestational age. Table S16: Kaplan–Meier estimates of time to first developmental disorder diagnosis by gestational age group. Table S17: Kaplan–Meier estimates of time to first special educational needs by gestational age group. Table S18: Cox proportional hazards model for time to first diagnosis of developmental disorder (complete case analysis). Table S19: Cox proportional hazards model for time t [file DMCN-68-263-s003.docx]

*Table S1 - ICD-10 codes and terms*

| **ICD-10 code** | **ICD-10 term** |
| --- | --- |
| G80 | Cerebral palsy |
| G80.0 | Spastic quadriplegic cerebral palsy |
| G80.1 | Spastic diplegic cerebral palsy |
| G80.2 | Spastic hemiplegic cerebral palsy |
| G80.3 | Dyskinetic cerebral palsy |
| G80.4 | Ataxic cerebral palsy |
| G80.8 | Other cerebral palsy |
| G80.9 | Cerebral Palsy, unspecified |
| G81 | Hemiplegia |
| G81.0 | Flaccid hemiplegia |
| G81.1 | Spastic hemiplegia |
| G81.9 | Hemiplegia unspecified |
| G82 | Paraplegia and tetraplegia |
| G82.0 | Flaccid paraplegia |
| G82.1 | Spastic paraplegia |
| G82.2 | Paraplegia |
| G82.3 | Flaccid tetraplegia |
| G82.4 | Spastic tetraplegia |
| G82.5 | Tetraplegia unspecified/quadriplegia NOS |
| G83 | other paralytic syndromes |
| G83.0 | Diplegia of upper limb |
| G83.1 | Monoplegia of lower limb |
| G83.2 | Monoplegia of upper limb |
| G83.3 | Monoplegia unspecified |
| G83.5 | Locked-in syndrome |
| G83.9 | Paralytic syndrome, unspecified |
| F82 | Specific developmental disorder of motor function |
| R27 | Other lack of coordination |
| R27.0 | Ataxia, unspecified |
| R27.8 | Other and unspecified lack of coordination |
| F06.7 | Mild cognitive disorder |
| F70 | Mild mental retardation |
| F70.0 | With the statement of no, or minimal, impairment of behaviour |
| F70.1 | Significant impairment of behaviour requiring attention or treatment |
| F70.8 | Other impairments of behaviour |
| F70.9 | Without mention of impairment of behaviour |
| F71 | Moderate mental retardation |
| F71.0 | With the statement of no, or minimal, impairment of behaviour |
| F71.1 | Significant impairment of behaviour requiring attention or treatment |
| F71.8 | Other impairments of behaviour |
| F71.9 | Without mention of impairment of behaviour |
| F72 | Severe mental retardation |
| F72.0 | With the statement of no, or minimal, impairment of behaviour |
| F72.1 | Significant impairment of behaviour requiring attention or treatment |
| F72.8 | Other impairments of behaviour |
| F72.9 | Without mention of impairment of behaviour |
| F73 | Profound mental retardation |
| F73.0 | With the statement of no, or minimal, impairment of behaviour |
| F73.1 | Significant impairment of behaviour requiring attention or treatment |
| F73.8 | Other impairments of behaviour |
| F73.9 | Without mention of impairment of behaviour |
| F78 | Other mental retardation |
| F78.0 | With the statement of no, or minimal, impairment of behaviour |
| F78.1 | Significant impairment of behaviour requiring attention or treatment |
| F78.8 | Other impairments of behaviour |
| F78.9 | Without mention of impairment of behaviour |
| F79 | Unspecified mental retardation |
| F79.0 | With the statement of no, or minimal, impairment of behaviour |
| F79.1 | Significant impairment of behaviour requiring attention or treatment |
| F79.8 | Other impairments of behaviour |
| F79.9 | Without mention of impairment of behaviour |
| F81 | Specific developmental disorders of scholastic skills |
| F81.0 | Specific reading disorder |
| F81.1 | Specific spelling disorder |
| F81.2 | Specific disorder of arithmetical skills |
| F81.3 | Mixed disorder of scholastic skills |
| F81.8 | Other developmental disorders of scholastic skills |
| F81.9 | Developmental disorder of scholastic skills, unspecified |
| Z55 | Problems related to education and literacy |
| Z55.0 | Illiteracy and low-level literacy |
| Z55.1 | Schooling unavailable and unattainable |
| Z55.2 | Failed examinations |
| Z55.3 | Underachievement in school |
| Z55.8 | Other problems related to education and literacy |
| Z55.9 | Problem related to education and literacy, unspecified |
| R41.8 | Other and unspecified symptoms and signs involving cognitive functions and awareness |
| F80 | Specific speech articulation disorder |
| F80.1 | Expressive language disorder |
| F80.2 | Receptive language disorder |
| F80.3 | Acquired aphasia with epilepsy [Landau-Kleffner] |
| F80.8 | Other developmental disorders of speech and language |
| F80.9 | Developmental disorder of speech and language, unspecified |
| F800 | Specific speech articulation disorder |
| F98.5 | Stuttering [stammering] |
| F98.6 | Cluttering |
| F84.4 | Overactive disorder associated with mental retardation and stereotyped movements |
| F90 | Hyperkinetic disorders |
| F90.0 | Disturbance of activity and attention - [inc. ADHD] |
| F90.1 | Hyperkinetic conduct disorder |
| F90.8 | Other hyperkinetic disorders |
| F90.9 | Hyperkinetic disorder, unspecified |
| F84 | Pervasive developmental disorders |
| F84.0 | Childhood autism |
| F84.1 | Atypical autism |
| F84.3 | Other childhood disintegrative disorder |
| F84.5 | Asperger's syndrome |
| F84.8 | Other pervasive developmental disorders |
| F84.9 | Pervasive developmental disorder, unspecified |
| F91 | Conduct disorders |
| F91.0 | Conduct disorder confined to the family context |
| F91.1 | Unsocialized conduct disorder |
| F91.2 | Socialized conduct disorder |
| F91.3 | Oppositional defiant disorder |
| F91.8 | Other conduct disorders |
| F91.9 | Conduct disorder, unspecified |
| F92 | Mixed disorders of conduct and emotions |
| F92.0 | Depressive conduct disorder |
| F92.8 | Other mixed disorders of conduct and emotions |
| F92.9 | Mixed disorder of conduct and emotions, unspecified |
| F93 | Emotional disorders with onset specific to childhood |
| F93.0 | Separation anxiety disorder of childhood |
| F93.1 | Phobic anxiety disorder of childhood |
| F93.2 | Social anxiety disorder of childhood |
| F93.3 | Sibling rivalry disorder |
| F93.8 | Other childhood emotional disorders |
| F93.9 | Childhood emotional disorder, unspecified |
| F94 | Disorders of social functioning with onset specific to childhood and adolescence |
| F94.0 | Elective mutism |
| F94.1 | Reactive attachment disorder of childhood |
| F94.2 | Disinhibited attachment disorder of childhood |
| F94.8 | Other childhood disorders of social functioning |
| F94.9 | Childhood disorder of social functioning, unspecified |
| F95 | Tic disorders |
| F95.1 | Chronic motor or vocal tic disorder |
| F95.2 | Combined vocal and multiple motor tic disorder [de la Tourette] |
| F95.8 | Other tic disorders |
| F95.9 | Tic disorder, unspecified |
| F98 | Other behavioural and emotional disorders with onset usually occurring in childhood and adolescence |
| F98.3 | Pica of infancy and childhood |
| F98.4 | Stereotyped movement disorders |
| F98.8 | Other specified behavioural and emotional disorders with onset usually occurring in childhood and adolescence |
| F98.9 | Unspecified behavioural and emotional disorders with onset usually occurring in childhood and adolescence |
| R45 | Symptoms and signs involving emotional state |
| R45.0 | Nervousness |
| R45.1 | Restlessness and agitation |
| R45.2 | Unhappiness |
| R45.3 | Demoralization and apathy |
| R45.4 | Irritability and anger |
| R45.5 | Hostility |
| R45.6 | Physical violence |
| R45.7 | State of emotional shock and stress, unspecified |
| R45.8 | Other symptoms and signs involving emotional state |
| Z55.4 | Educational maladjustment and discord with teachers and classmates |
| F98.2 | Feeding disorder of infancy and childhood |
| R63.3 | Feeding difficulties and mismanagement |
| G47.3 | Sleep apnoea |
| H53.4 | Visual field defects |
| H54 | Visual impairment including blindness (binocular or monocular) |
| H54.0 | Blindness, binocular |
| H54.1 | Severe visual impairment, binocular |
| H54.2 | Moderate visual impairment, binocular |
| H54.4 | Blindness, monocular |
| H54.5 | Severe visual impairment, monocular |
| H54.6 | Moderate visual impairment, monocular |
| H54.7 | Unspecified visual loss |
| H54.9 | Unspecified visual impairment (binocular) |
| H90 | Conductive and sensorineural hearing loss |
| H91.3 | Deaf mutism, not elsewhere classified |
| H90.6 | Mixed conductive and sensorineural hearing loss, bilateral |
| H90.7 | Mixed conductive and sensorineural hearing loss, unilateral with unrestricted hearing on the contralateral side |
| H90.8 | Mixed conductive and sensorineural hearing loss, unspecified |
| H91.0 | Ototoxic hearing loss |
| H90.3 | Sensorineural hearing loss, bilateral |
| H90.4 | Sensorineural hearing loss, unilateral with unrestricted hearing on the contralateral side |
| H90.5 | Sensorineural hearing loss, unspecified |
| F83 | Mixed specific developmental disorders |
| F88 | Other disorders of psychological development |
| F88X | Other disorders of psychological development |
| F89 | Unspecified disorder of psychological development |
| F89X | Unspecified disorder of psychological development |
| F98.0 | Nonorganic enuresis |
| F98.1 | Nonorganic encopresis |
| R62.0 | Delayed milestone |
| R62.9 | Lack of expected normal physiologic development unspec |

Table S2 - Clinical terms version 3 (CTV3) “Read” codes and terms

| **code** | **term** |
| --- | --- |
| F1370 | (Athetoid cerebral palsy) or (Vogt's dis: [ophth][neurol]) |
| P22z. | (Cereb hypopl)(red def brain NOS)([agen][hypopl] brain NEC) |
| F233. | (Congenital monoplegia) or (congenital spastic foot) |
| F24yz | (Other paralytic syndromes NOS) or (specified palsy NEC) |
| XE183 | (Paralytic syndromes NOS) or (diplegia) or (monoplegia) |
| Fyu9. | [X]Cerebral palsy and other paralytic syndromes |
| Fyu90 | [X]Other infantile cerebral palsy |
| Xa0lM | Ataxic cerebral palsy |
| F23y0 | Ataxic infantile cerebral palsy |
| XE2Q7 | Athetoid cerebral palsy |
| F1371 | Athetosis: [double] or [congenital] |
| F2B2. | Bilateral spastic cerebral palsy |
| Xab3R | Bilateral spastic cerebral palsy |
| F2Bz. | Cerebral palsy |
| XE2Q8 | Cerebral palsy |
| F232. | Cerebral palsy &/or congenital quadriplegia |
| XE181 | Cerebral palsy (&/or infantile) &/or infantile hemiplegia |
| F2B.. | Cerebral palsy (syn: "CP- Cerebral palsy") |
| F2By. | Cerebral palsy (synonyms: CP- Cerebral palsy) |
| XE2Q9 | Cerebral palsy with spastic diplegia (syn: spastic diplegic cerebral palsy) |
| F2301 | Cerebral palsy with spastic diplegia (synonyms: "Spastic diplegic cerebral palsy") |
| X00Eo | Cerebral palsy with spastic tetraparesis |
| XE2se | Cerebral palsy with spastic tetraplegia |
| X00Eq | Cerebral palsy with spastic/ataxic diplegia (synonoyms: 'ataxic diplegic ceral palsy' |
| XaBE2 | Cerebral palsy, not congenital or infantile, acute |
| G669. | Cerebral palsy, not congenital or infantile, acute |
| F23y6 | Choreoathetoid cerebral palsy (synonyms: choreo-athetoic cerebral palsy) |
| XaadE | Choreoathetoid cerebral palsy (synonyms: choreo-athetotic cerebral palsy') |
| X00Ey | Congenital apraxia |
| XaB4P | Congenital athetosis |
| XM1Pu | Congenital cerebral palsy |
| F23.. | Congenital cerebral palsy (& spastic) |
| .F32Z | Congenital cerebral palsy NOS |
| F23z. | Congenital cerebral palsy NOS |
| XM1Px | Congenital diplegia |
| F230z | Congenital diplegia NOS |
| Y6151 | Congenital hemiplegia |
| XE15V | Congenital monoplegia |
| Xa0lI | Congenital non-progressive ataxia |
| F2300 | Congenital paraplegia |
| XM1Pw | Congenital quadriplegia |
| XM1Pv | Congenital spastic cerebral palsy |
| X00Et | Congenital spastic foot |
| XM03s | Diplegia |
| F242. | Diplegia of upper limbs |
| XE15M | Double athetosis |
| X00Ex | Dysequilibrium syndrome (syn DES - dysequilibrium syndrome) |
| F23y3 | Dyskinetic cerebral palsy |
| X00Eu | Dyskinetic cerebral palsy |
| X00Ew | Dystonic/rigid cerebral palsy |
| F220. | Flaccid hemiplegia |
| F2410 | Flaccid paraplegia |
| F2400 | Flaccid tetraplegia |
| XaaVG | Gross Motor Function Classification System for Cerebral Palsy |
| XaaVI | Gross Motor Function Classification System for Cerebral Palsy level finding (synonym: GMFCS (Gross Motor Function Classification System) for cerebral palsy level finding) |
| XaaVJ | Gross Motor Function Classification System for Cerebral Palsy level I (syn: GMFCS for CP level 1) |
| XaaVK | Gross Motor Function Classification System for Cerebral Palsy level II (synonym: GMFCS…for cerebral palsy levelII) |
| XaaWD | Gross Motor Function Classification System for Cerebral Palsy level III (synonym: "GMFCS..for cerebral palsy level III") |
| XaaWE | Gross Motor Function Classification System for Cerebral Palsy level IV (synonym: "GMFCS..for cerebral palsy level IV") |
| XaaWF | Gross Motor Function Classification System for Cerebral Palsy level V |
| XE15T | Hemiplegia |
| F22z. | Hemiplegia NOS |
| F23y1 | Hypotonic cerebral palsy |
| X00Em | Infantile cerebral palsy (synonyms "ICP - infantile cerebral palsy") |
| Y7227 | Infantile cerebral palsy NOS |
| F231. | Infantile hemiplegia (syn: 'Hemiplegic cerebral palsy') |
| F234. | Infantile hemiplegia NOS |
| X50In | Keratoderma with mental retardation and spastic paraplegia |
| Xa0fs | Left hemiplegia |
| F222. | Left: [hemiplegia] or [sided weakness] |
| XaeUO | Locked in syndrome |
| Y2b6a | Mixed cerebral palsy |
| XM03p | Monoplegia |
| F243. | Monoplegia of lower limb |
| F244. | Monoplegia of upper limb |
| F245. | Monoplegia unspecified |
| X00Es | Monoplegic cerebral palsy affecting lower limb |
| X00Er | Monoplegic cerebral palsy affecting upper limb |
| F23y. | Other congenital cerebral palsy |
| F23yz | Other infantile cerebral palsy NOS |
| F24.. | Other paralytic syndromes |
| XE15X | Other paralytic syndromes NOS |
| F24y. | Other specified paralytic syndromes |
| F24y. | Other specified paralytic syndromes |
| XM03m | Paralysis |
| F24z. | Paralysis NOS |
| 1B33. | Paralysis present |
| Xa3fA | Paralytic syndromes |
| F241. | Paraplegia |
| X00Ep | Paraplegia - congenital |
| F240. | Quadriplegia |
| XaELO | Residual hemiplegia |
| Xa0fr | Right hemiplegia |
| F223. | Right: [hemiplegia] or [sided weakness] |
| F23y2 | Spastic cerebral palsy |
| X00En | Spastic cerebral palsy |
| Xa1kT | Spastic diplegia |
| XE15U | Spastic hemiplegia |
| XaYfK | Spastic hemiplegic cerebral palsy |
| F2411 | Spastic paraplegia |
| X76je | Spastic quadriplegia |
| XaYgp | Spastic quadriplegic cerebral palsy |
| F2401 | Spastic tetraplegia |
| XaB3p | Specified palsy NEC |
| E2F4. | (Aprax, dev) or (clums syn) or (dysprax syn) or (co-ord dis) |
| R0130 | [D]Ataxia NOS |
| R0132 | [D]Dysgraphia |
| XaO46 | [D]Fine motor skills development delay |
| XaIsc | [D]Gross motor skills development delay |
| R013z | [D]Incoordination NOS |
| R013. | [D]Lack of coordination |
| R0131 | [D]Muscular incoordination |
| Eu82. | [X]Specific developm disord motor funct (& [named variants]) |
| Ub1Ti | Acquired choreiform dyspraxia |
| XaVzu | Apraxia |
| XM1MS | Apraxia, developmental |
| X76q8 | Arms ataxic |
| X76q7 | Ataxia |
| XM0z3 | Ataxia [D] |
| X76q4 | Bumps into things |
| XM0qi | Cerebellar ataxia |
| Xa7PA | Clumsiness |
| XaBBv | Clumsiness -motor delay |
| X75Za | Constructional dyspraxia |
| Xa8Kq | Coordination problem |
| Y25e1 | DCD - developmental coordination disorder |
| Ub1Tg | Developmental articulatory dyspraxia |
| XE1Z5 | Developmental disorder of motor function |
| Ub1Th | Developmental verbal dyspraxia |
| X76q6 | Drops things |
| X76qC | Dysdiadochokinesis |
| Xa6tj | Dysdiadochokinesis present |
| XaVzt | Dyspraxia |
| X76qA | Finger-finger test abnormal |
| X76q9 | Finger-nose test abnormal |
| X76q5 | Fumbles with things |
| X76pr | Gait apraxia |
| X75ZZ | Gestural apraxia |
| X76qE | Heel-shin test abnormal |
| Ub1Tk | Ideational dyspraxia |
| Ub1Tl | Ideomotor dyspraxia |
| Xa1bq | Incoordination |
| XM0z2 | Incoordination [D] |
| XE0rt | Incoordination symptom |
| 1B5Z. | Incoordination symptom NOS |
| X76qD | Legs ataxic |
| Ub1Tj | Mixed acquired dyspraxia |
| XaXCG | Motor developmental delay |
| Xa1br | Muscular incoordination |
| XaItt | Not yet sitting |
| XaItu | Not yet standing |
| XaIts | Not yet walking |
| XM1Wn | O/E - ataxia |
| 29L9. | O/E - clumsy |
| 29LZ. | O/E - coordination NOS |
| 29L7. | O/E - dysdiadochokinesia present |
| 29L8. | O/E - generally unsteady |
| 29L3. | O/E - legs ataxic |
| 29L6. | O/E - past pointing present |
| XaKK9 | Oculomotor apraxia - Cogan type |
| Xa867 | Oculomotor dyspraxia |
| Ub1Ta | Oral dyspraxia |
| XM1Au | Other and unspecified lack of coordination |
| X76qB | Past pointing |
| Xa6tl | Past pointing present |
| Xa35E | Poor coordination |
| XE2bB | Specific developmental disorder of motor function |
| Xa0to | Speech and language dyspraxias |
| Ub1To | Tongue tip dyspraxia |
| X76qF | Truncal ataxia |
| E30.. | (Mild mental retard (& [feeble-mind][moron])) or (educ subn) |
| ZV400 | [V]Problems with learning |
| Eu7z. | [X] (Unspec mental retard) or (mental defic [& subnorm] NOS) |
| Eu700 | [X]Mild mental retardation with the statement of no, or minimal, impairment of behaviour |
| Eu70z | [X]Mild mental retardation without mention of impairment of behaviour |
| Eu70y | [X]Mild mental retardation, other impairments of behaviour |
| Eu701 | [X]Mild mental retardation, significant impairment of behaviour requiring attention or treatment |
| Eu71y | [X]Mod retard oth behav impair |
| Eu710 | [X]Moderate mental retardation with the statement of no, or minimal, impairment of behaviour |
| Eu71z | [X]Moderate mental retardation without mention of impairment of behaviour |
| Eu711 | [X]Moderate mental retardation, significant impairment of behaviour requiring attention or treatment |
| Eu7y. | [X]Other mental retardation |
| Eu7y0 | [X]Other mental retardation with the statement of no, or minimal, impairment of behaviour |
| Eu7yz | [X]Other mental retardation without mention of impairment of behaviour |
| Eu7yy | [X]Other mental retardation, other impairments of behaviour |
| Eu7y1 | [X]Other mental retardation, significant impairment of behaviour requiring attention or treatment |
| Eu730 | [X]Profound mental retardation with the statement of no, or minimal, impairment of behaviour |
| Eu73z | [X]Profound mental retardation without mention of impairment of behaviour |
| Eu73y | [X]Profound mental retardation, other impairments of behaviour |
| Eu731 | [X]Profound mental retardation, significant impairment of behaviour requiring attention or treatment |
| Eu720 | [X]Severe mental retardation with the statement of no, or minimal, impairment of behaviour |
| Eu72z | [X]Severe mental retardation without mention of impairment of behaviour |
| Eu72y | [X]Severe mental retardation, other impairments of behaviour |
| Eu721 | [X]Severe mental retardation, significant impairment of behaviour requiring attention or treatment |
| XE1a2 | [X]Unspecified mental retardation |
| Eu7z0 | [X]Unspecified mental retardation with the statement of no, or minimal, impairment of behaviour |
| Eu7zz | [X]Unspecified mental retardation without mention of impairment of behaviour |
| Eu7zy | [X]Unspecified mental retardation, other impairments of behaviour |
| Eu7z1 | [X]Unspecified mental retardation, significant impairment of behaviour requiring attention or treatment |
| X75wy | Below average intellect |
| Xa1aW | Borderline mental retardation |
| XaAyZ | Difficulty analysing information |
| Xa8LF | Difficulty comprehending concept of danger |
| XacBU | Difficulty comprehending language |
| XacBN | Difficulty comprehending speech |
| XacBc | Difficulty comprehending written material |
| XaA3B | Difficulty making considered choices |
| XaA2O | Difficulty performing logical sequencing |
| XaAyV | Difficulty processing information |
| XaA2L | Difficulty reasoning |
| Xac3m | Difficulty understanding written language |
| XaA2d | Difficulty using arithmetic reasoning |
| XaA2T | Difficulty using decision-making strategies |
| XaA2h | Difficulty using verbal reasoning |
| XaA2Y | Difficulty using visuospatial reasoning |
| XacBd | Does comprehend written material |
| XacBW | Does not comprehend language |
| XacBP | Does not comprehend speech |
| XacBe | Does not comprehend written material |
| Xac3k | Does not understand verbal language |
| Xac3o | Does not understand written language |
| X75xn | Impaired ability to learn new material |
| Ua189 | Impaired cognition |
| XacF6 | Intellectual development disorder of unknown aetiology |
| 13Z3. | Intelligence quotient low |
| XaJW7 | Learning disabilities administration status |
| XaL3Q | Learning disabilities annual health assessment |
| XaJsd | Learning disabilities health action plan completed |
| XaJW9 | Learning disabilities health action plan declined |
| XaJW8 | Learning disabilities health action plan offered |
| XaJWA | Learning disabilities health action plan reviewed |
| XaJmb | Learning disabilities health assessment |
| XaPx2 | Learning disability health examination |
| X75x1 | Low intelligence |
| .6664 | Mental handicap problem |
| 6664 | Mental handicap problem |
| E3... | Mental retardation |
| E3z.. | Mental retardation NOS |
| XaDki | Mental retardation, congenital heart disease, blepharophimosis, blepharoptosis and hypoplastic teeth |
| Xaagi | Mild cognitive impairment |
| Eu816 | Mild learning disability |
| XaREt | Mild learning disability |
| XE2a3 | Mild mental retardation, IQ in range 50-70 |
| Xaagj | Moderate cognitive impairment |
| Eu814 | Moderate learning disability |
| XaQZ3 | Moderate learning disability |
| .E512 | Moderate mental retardation, IQ in range 35-49 |
| E310. | Moderate mental retardation, IQ in range 35-49 |
| Eu71. | Moderate mental retardation, IQ in range 35-49 |
| Xa00k | Moderate mental retardation, IQ in range 35-49 |
| 918e. | On learning disability register |
| XaKYb | On learning disability register |
| Y1642 | Other Mental Disability |
| E31.. | Other specified mental retardation |
| E31z. | Other specified mental retardation NOS |
| XaREu | Profound learning disability |
| Eu817 | Profound learning disability |
| Y9981 | Profound mental retardation |
| E312. | Profound mental retardation with IQ less than 20 |
| Eu73. | Profound mental retardation with IQ less than 20 |
| Xa01E | Profound mental retardation with IQ less than 20 |
| XaLKE | Seen in learning disabilities clinic |
| Xaagk | Severe cognitive impairment |
| XaQZ4 | Severe learning disability |
| YA841 | Severe learning disability |
| Eu815 | Severe learning disability |
| .E513 | Severe mental retardation, IQ in range 20-34 |
| E311. | Severe mental retardation, IQ in range 20-34 |
| Eu72. | Severe mental retardation, IQ in range 20-34 |
| Xa00l | Severe mental retardation, IQ in range 20-34 |
| Xabk1 | Significant learning disability |
| Ua187 | Slow learner |
| XaaiS | Specific learning disability |
| Eu818 | Specific learning disability |
| XaAyY | Unable to analyse information |
| Xa8LE | Unable to comprehend concept of danger |
| XaA3A | Unable to make considered choices |
| XaA2P | Unable to perform logical sequencing |
| XaAyU | Unable to process information |
| XaA2K | Unable to reason |
| XaA2c | Unable to use arithmetic reasoning |
| XaA2S | Unable to use decision-making strategies |
| XaA2g | Unable to use verbal reasoning |
| XaA2X | Unable to use visuospatial reasoning |
| XE1bm | (Disturbance in learning) or (development delay - NOS) |
| XE1bi | (Dyslexia: (& [developmental]) or reading delay |
| Eu81y | [X]Devel disord scholastic skills: [oth][expressive writing] |
| Eu81z | [X]Developmental disorder of scholastic skills, unspecified |
| XE1a9 | [X]Other developmental disorders of scholastic skills |
| Eu81. | [X]Specific developmental disorders of scholastic skills |
| Eu812 | [X]Specific disorder of arithmetical skills |
| Eu811 | [X]Specific spelling disord (& [retardn, no reading disord]) |
| E2Dy2 | Academic underachievement disorder |
| Xa0tp | Agraphia |
| E2F01 | Alexia |
| 13ZK. | Child with special educational needs |
| Ub1TD | Deep dysgraphia |
| Ub1TH | Deep dyslexia |
| X00TL | Developmental disorder of scholastic skill |
| E2F02 | Developmental dyslexia |
| XaBmg | Difficulty reading |
| XaAzQ | Difficulty writing |
| E2F1. | Dyscalculia |
| Ub1TC | Dysgraphia |
| Ub00K | Dyslexia |
| Xa0ER | Educationally subnormal |
| Ub0ih | Intellectual functioning disability |
| 13Z4E | Learning difficulties |
| Eu813 | Mixed disorder of scholastic skills |
| E2F2. | Other specific learning difficulty |
| Ub1TG | Paragraphia |
| Ub1TE | Phonological dysgraphia |
| Ub1TI | Phonological dyslexia |
| XaDyv | Provision of special educational needs nursery |
| E2F00 | Reading disorder unspecified |
| Xa3HI | Severely educationally subnormal |
| Y1790 | Specific arithmetical disorder |
| E2F0. | Specific reading disorder |
| E2F0z | Specific reading disorder NOS |
| XE1a8 | Specific spelling disorder |
| Ub1TF | Surface dysgraphia |
| Ub1TJ | Surface dyslexia |
| XaBmf | Unable to read |
| XaAzP | Unable to write |
| X75x5 | Difficulty solving problems |
| X75x7 | Difficulty making plans |
| X75x8 | Difficulty making decisions |
| XaCy7 | Unable to process information accurately |
| XaCy8 | Difficulty processing information accurately |
| XaCyA | Unable to process information at normal speed |
| XaCyB | Difficulty processing information at normal speed |
| Xa2Ve | Impairment of working memory |
| E2F3. | (Develop disord: [language][speech]) or (articulatn defect) |
| XE1bk | (Developm aphasia) or (delayed speech) or (speech delay) |
| E2F30 | (Developmental aphasia) or (word deafness) |
| XaO47 | [D]Communication skills development delay |
| XaKYj | [D]Dysfluency |
| ZV401 | [V]Problems with communication, including speech |
| Eu80y | [X] (Other developmental disorders of speech and language) or (lisping) |
| Eu80z | [X] Developmental disorder of speech &/or language, unspecified or NOS |
| Eu801 | [X] Expressive language disorder: [developmental dysphasia, expressive type] or [developmental aphasia, expressive type] |
| Eu802 | [X] Receptive language disorder (& [congenit auditory imperception] or [development dysphasia, receptive] or [development Wernicke's aphasia] or [word deafness] or [development aphasia, receptive]) |
| Eu800 | [X] Specific speech articulation disorder (& [developmental (& phonological)] or [dyslalia] or [functional] or [lalling]) |
| XM19U | [X] Speech disturbances, not elsewhere classified |
| XE1a7 | [X]Developmental disorder of speech and language, unspecified |
| XE1a6 | [X]Other developmental disorders of speech and language |
| XE1a5 | [X]Receptive language disorder |
| Ub1T2 | Acoustic analysis deficit |
| XabxE | Acquired dysarthria |
| Xa0tr | acquired dysphasias |
| XacKz | Acquired language comprehension impairment |
| Ub1Sf | Acquired language disorder |
| XacKv | Acquired receptive language impairment |
| Ub1TL | Anarthria |
| Ub1Sk | Anomia |
| Ub1Ss | Anterior dysphasia |
| Ub1Sg | Aphasia |
| Ub1TZ | Apraxia of speech |
| Xabxq | Articulatory defect due to conductive hearing loss |
| Ub1Td | Articulatory dyspraxia |
| Ub1Uf | Auditory processing disorder |
| XacFo | Backing of sounds |
| Ub1TU | Bradyarthria |
| Ub1Si | Broca's dysphasia |
| Ub1TQ | Cerebellar dysarthria |
| XacFq | Cognitive communication disorder |
| Ub1Sp | Conduction dysphasia |
| Ub1Ua | Congenital auditory imperception |
| X00F0 | Congenital dysarthria |
| X00F1 | Congenital dysphasia |
| X00F2 | Congenital expressive dysphasia |
| X00F3 | Congenital receptive dysphasia |
| XacFp | De-affrication |
| Ub1US | Delayed pre-verbal development |
| XE1Z4 | Developmental aphasia |
| Ub1Tg | Developmental articulatory dyspraxia |
| Ub1UZ | Developmental dysphasia |
| XE1a4 | Developmental expressive language disorder |
| XacL0 | Developmental language comprehension impairment |
| Ub1UQ | Developmental language delay |
| Ub1UR | Developmental language disorder |
| Ub1UG | Developmental language impairment |
| Ub1Tf | Developmental motor speech disorder |
| XacKx | Developmental receptive language impairment |
| Ub1UX | Developmental semantic impairment |
| XE1a3 | Developmental speech articulation disorder |
| Ub1U0 | Developmental speech disorder |
| Ub1UW | Developmental syntactic impairment |
| XaCar | Discourse difficulties |
| XE1Z3 | Disorder of speech and language development |
| Ub1TM | Dysarthria |
| Ub1TV | Dysarthria of velopharynx |
| Ub1TT | Dysarthriapneumophonia |
| Ub1S3 | Dysfluency |
| E2F31 | Dyslalia |
| Ub1Tm | Dyspraxia of velopharynx |
| Ub1Sy | Efferent motor dysphasia |
| Ub1Sx | Expressive dysphasia |
| Ub1UM | Expressive language delay |
| Ub1UL | Expressive language disorder |
| Ub1UI | Expressive language impairment |
| Ub1TR | Extrapyramidal dysarthria |
| Ub1TO | Flaccid dysarthria |
| Ub1Sm | Fluent dysphasia |
| Ub1Sl | Frontal dynamic dysphasia |
| XacFn | Fronting of sounds |
| Ub1Sq | Global dysphasia |
| Ub1T3 | Grapheme-phoneme conversion deficit |
| Ub1Te | Immature articulatory praxis |
| Ub1U7 | Immature sound system |
| Ub0hr | Impairment of speech form |
| Ub0hq | Impairment of voice production |
| X00TK | Language development disorder |
| Ub1T5 | Language disorder associated with right hemisphere damage |
| Ub1T7 | Language disorder associated with thought disorder |
| Ub1UH | Language impairment |
| Ub1TB | Language-related cognitive disorder |
| Ub1Ud | Lexical syntactic disorder |
| Ub1U9 | Limited sound system |
| Ub1TP | Mixed dysarthria |
| Ub1Su | Mixed dysphasia |
| Ub1Sr | Mixed transcortical dysphasia |
| Ub1TK | Motor speech disorder |
| Ub1SD | Neurogenic stammering |
| Ub1UA | Non-contrastive sound system |
| Ub1Sh | Non-fluent dysphasia |
| XaItr | Not yet speaking |
| XaIUM | O/E - speech delay |
| Ub1Tc | Oral-verbal dyspraxia |
| Ub1T4 | Phoneme-grapheme conversion deficit |
| Ub1U2 | Phonological delay |
| Ub1U3 | Phonological disorder |
| Ub1U4 | Phonological programming deficit |
| Ub1UK | Phonological syntactic disorder |
| Ub1St | Posterior dysphasia |
| Ub1T9 | Post-traumatic mutism |
| Ub1TS | Pyramidal dysarthria |
| Ub1Sw | Receptive dysphasia |
| Ub1UO | Receptive language delay |
| Ub1UJ | Receptive language impairment |
| Ub1UU | Restricted expressive language development |
| Ub1UT | Restricted language development |
| Ub1UV | Restricted receptive language development |
| Ub1U8 | Restricted sound system |
| Ub1Sv | Semantic dysphasia |
| Ub1T1 | Semantic impairment |
| Ub1Ue | Semantic-pragmatic disorder |
| Ub1Tt | Semantic-pragmatic impairment |
| XaD2v | Sociolinguistic difficulties |
| Ub1TN | Spastic dysarthria |
| Ub1UY | Specific language impairment |
| XaaaS | Specific speech impairment |
| X00Tb | Speech and language disorder |
| Xa0tq | Speech and phonology impairments |
| Ub1U6 | Speech delay |
| Ub0hp | Speech impairment |
| E2F3z | Speech or language developmental disorder NOS |
| Ub1Sz | Subcortical aphasia |
| Xa4Pz | Syllable deletion |
| Ub1T0 | Syntactic impairment |
| Ub1Sj | Transcortical motor dysphasia |
| Ub1So | Transcortical sensory dysphasia |
| Ub1Tn | Velar dyspraxia |
| Ub1Tb | Verbal dyspraxia |
| Ub1UP | Word finding difficulty |
| Ua1XX | Unable to concentrate |
| X760c | Reduced concentration |
| X760d | Reduced concentration span |
| XSJK7 | Poor concentration |
| E2E1. | Hyperkinesis with developmental delay |
| XE2Q6 | Attention deficit hyperactivity disorder |
| Eu844 | [X]Overactive disorder associated with mental retardation and stereotyped movements |
| 1P00. | Hyperactive behaviour |
| E2E.. | Childhood hyperkinetic syndrome |
| E2E0. | Child attention deficit disorder |
| E2E00 | Attention deficit without hyperactivity |
| E2E01 | Attention deficit hyperactivity disorder |
| E2E0z | Child attention deficit disorder NOS |
| E2E2. | Hyperkinetic conduct disorder |
| E2Ey. | Other hyperkinetic manifestation |
| E2Ez. | Hyperkinetic syndrome NOS |
| Eu90. | overactive child syndrome |
| Eu900 | [X]Disturbance of activity and attention |
| Eu901 | Hyperkinetic conduct disorder |
| Eu902 | Deficits in attention motor control and perception |
| Eu90y | [X]Other hyperkinetic disorders |
| Eu90z | [X]Hyperkinetic disorder, unspecified |
| E2C3. | Impulse control disorder NEC |
| E2C30 | Impulse control disorder, unspecified |
| E2C3z | Impulse control disorder NOS |
| Y2916 | Autistic spectrum disorder - Gluten-casein intolerance checklist |
| Y2917 | Autistic spectrum disorder - Autism checklist |
| .E2Z3 | Autistic spectrum disorder (synonyms: pervasive developmental disorder, autistic continuum, PPD) |
| E140. | Childhood autism (syn: Kanner's syndrome, autistic disorder, infantile autism, infantile psychosis) |
| E1400 | Active infantile autism |
| E1401 | Residual infantile autism |
| E140z | Infantile autism NOS |
| E141. | Disintegrative psychosis (synonyms: Heller's syndrome) |
| E1410 | Active disintegrative psychoses |
| E141z | Disintegrative psychosis NOS |
| Eu84. | Autistic spectrum disorder (syn: pervasive developmental disoder, autistic continuum, PPD - dervasive developmental disorder) |
| Eu840 | Childhood autism (syn: Kanner's syndrome, autistic disorder, infantile autism, infantile psychosis) |
| Eu841 | Atypical autism (syn: Atypical autistic syndrome) |
| Eu845 | Asperger syndrome ~(synonyms: Schizoid disorder of childhood) |
| Eu84y | [X]Other pervasive developmental disorders |
| Eu84z | [X]Pervasive developmental disorder, unspecified (syn, autistic spectrum disorder) |
| Ub1Tr | Autistic spectrum disorder (synonocms: pervasive developmental disorder, autistic continuum, PPD - pervasive developmental disorder) |
| Ub1Ts | Idiot savant (synonyms: autistic spectrum disorder with isolated skills) |
| Ub1Tw | Persistent developmental avoidance (synonyms: PDA) |
| X00TM | Autistic spectrum disorder |
| X00TN | Atypical autism (synonyms: 'atypical autistic syndrome') |
| X00TP | Asperger syndrome (synonyms: Schizoid disorder of childhood) |
| XaesO | Pathological demand avoidance (synonyms: PDA, Newson's syndrome) |
| XE1aA | [X]Other childhood disintegrative disorder |
| XE2v2 | Childhood autism (synonyms: Kanner's syn, Autistic disorder, Infantile autism, Infantile psychosis |
| E1411 | Residual disintegrative psychoses |
| E2D3. | Childhood and adolescent relationship problem |
| E2D30 | Sibling jealousy |
| E2D3z | Childhood and adolescent relationship problem NOS |
| Xa1ai | Behavioural disability |
| XaLQL | Emotional behaviour disability |
| ZV40. | [V]Mental and behavioural problems |
| ZV403 | [V]Other behavioural problems |
| ZV404 | [V]Pathological sick role |
| ZV40y | [V]Other specified mental or behavioural problem |
| ZV40z | [V]Unspecified mental or behavioural problem |
| E2Dy1 | Childhood or adolescent identity disorder |
| X00TT | Adolescent - emotional problem |
| Xa1b6 | Childhood overanxious disorder |
| XE1Yv | Childhood emotional disorder |
| XE1Yw | Childhood and adolescence disturbance of unhappiness |
| XE2bC | Sibling rivalry disorder |
| XaO45 | [D]Social skills development delay |
| Eu843 | [X]Child disinteg dis:[oth][dement infantalis (& named var)] |
| Eu8y. | [X]Disord psychological developm: [other] or [devel agnosia] |
| E292. | Adjustment reaction with predominant disturbance of other emotions |
| E2920 | Separation anxiety disorder |
| E292y | Adjustment reaction with mixed disturbance of emotion |
| E292z | Adjustment reaction with disturbance of other emotion NOS |
| E294. | Adjustment reaction with mixed disturbance of emotion and conduct |
| E2C0. | Aggressive unsocial conduct disorder |
| E2C00 | Aggressive outburst |
| E2C1z | Nonaggressive unsocial conduct disorder NOS |
| E2C2. | Conduct disorder - socialised |
| E2C2z | Socialised conduct disorder NOS |
| E2C4. | Childhood disorder of conduct and emotion |
| E2C40 | Neurotic delinquency |
| E2C4z | Mixed disturbance of conduct and emotion NOS |
| E2Cy. | Other conduct disturbances |
| E2Cyz | Other conduct disturbances NOS |
| E2Cz. | Unspecified disturbance of conduct |
| E2Cz0 | Juvenile delinquency unspecified |
| E2Czz | Disturbance of conduct NOS |
| E2D0. | Disturbance of anxiety and fearfulness in childhood and adolescence |
| E2D00 | Childhood overanxious disorder |
| E2D01 | Childhood and adolescent fearfulness disturbance |
| E2D0z | Disturbance of anxiety and fearfulness in childhood and adolescence NOS |
| E2Dy. | Other childhood and adolescent emotional problems |
| E2Dy0 | Oppositional defiant disorder |
| E2Dyz | Other childhood and adolescent emotional problems NOS |
| Eu9.. | [X]Behavioural and emotional disorders with onset usually occurring in childhood and adolescence |
| Eu91. | [X]Conduct disorders |
| Eu910 | [X]Conduct disorder confined to the family context |
| Eu911 | Conduct disorder - unsocialised |
| Eu913 | Oppositional defiant disorder |
| Eu91y | [X]Other conduct disorders |
| Eu920 | Depressive conduct disorder |
| Eu92z | Mixed disturbance of conduct and emotion NOS |
| Eu93. | [X]Emotional disorders with onset specific to childhood |
| Eu930 | [X]Separation anxiety disorder of childhood |
| Eu931 | Childhood phobic anxiety disorder |
| Eu93z | [X]Childhood emotional disorder, unspecified |
| Eu94y | [X]Other childhood disorders of social functioning |
| Eu94z | [X]Childhood disorder of social functioning, unspecified |
| Eu9y. | [X]Other behavioural and emotional disorders with onset usually occurring in childhood and adolescence |
| Eu9yy | [X]Other specified behavioural and emotional disorders with onset usually occurring in childhood and adolescence |
| Eu9yz | [X]Unspecified behavioural and emotional disorders with onset usually occurring in childhood and adolescence |
| R034C | [D]Social skills development delay |
| XE1aD | Childhood avoidant disorder |
| Eu9y2 | Psychogenic feeding disorder of infancy and childhood |
| G56z. | Conduction disorders unspecified |
| XE1aE | [X]Other childhood emotional disorders |
| XE1Yr | Disturbance of conduct NEC |
| XE1Yt | Unsocial childhood truancy |
| XE1Z2 | Childhood and adolescent emotion disorder NOS |
| E2C0z | Aggressive unsocial conduct disorder NOS |
| E2C20 | Socialised childhood truancy |
| E2C23 | Group delinquency |
| E2C34 | Intermittent explosive disorder |
| E2C35 | Isolated explosive disorder |
| Eu9y3 | Pica of infancy and childhood |
| X00TR | Behavioural and emotional disorder with onset in childhood |
| X00TU | Childhood conduct disorder |
| X00TV | Conduct disorder - in family context |
| X00TW | Conduct disorder - unsocialised |
| Xac0T | Behavioural, emotional and social difficulties |
| Xac0U | Emotional behavioural difficulties |
| XE1Yx | Childhood and adolescent disturbance with sensitivity |
| XE1Yy | Childhood and adolescent disturbance with shyness |
| XE1Yz | Childhood and adolescent disturbance with introversion |
| XE1Z1 | Childhood and adolescent sensitivity disturbance NOS |
| E2755 | Non-organic infant feeding disturbance |
| XE1Ys | Nonaggressive unsocial conduct disorder |
| 163Z. | Feeding problem NOS |
| R033. | [D]Feeding difficulties and mismanagement |
| R0330 | [D]Feeding problem in infant |
| R033z | [D]Feeding difficulties NOS |
| XabAP | Developmental dysphagia |
| 163.. | Feeding problem symptom |
| XSJFL | Feeding problem in child |
| 1932. | Difficulty chewing |
| 193.. | Chewing symptoms |
| 193Z. | Chewing symptom NOS |
| Xa4JE | Unable to chew |
| Xa4JG | Does not chew |
| XaaxN | Developmental chewing difficulty |
| XaaxT | Acquired chewing difficulty |
| Xa4KC | Unable to suck |
| Xa4KE | Does not suck |
| Xa4KF | Difficulty sucking |
| XacSD | Developmental delay in feeding |
| R0051 | [D]Insomnia with sleep apnoea |
| X0083 | Sleep apnoea |
| X0084 | Obstructive sleep apnoea |
| X0085 | Central sleep apnoea |
| X0086 | Mixed sleep apnoea |
| X0087 | Alveolar sleep apnoea |
| XaEGP | [D]Sleep apnoea syndrome |
| XE2nU | [D]Hypersomnia with sleep apnoea |
| 1B75. | Blindness |
| F49.. | Impaired vision (& [blindness &/or low] or [partial sight]) |
| F4900 | Unspecified blindness both eyes |
| F4901 | Both eyes total visual impairment |
| F4902 | Better eye: near total visual impairment, Lesser eye: unspecified |
| F4903 | Better eye: near total visual impairment, Lesser eye: total visual impairment |
| F4904 | Better eye: near total visual impairment, Lesser eye: near total visual impairment |
| F4905 | Better eye: profound visual impairment, Lesser eye: unspecified |
| F4906 | Better eye: profound visual impairment, Lesser eye: total visual impairment |
| F4907 | Better eye: profound visual impairment, Lesser eye: near total visual impairment |
| F4908 | Better eye: profound visual impairment, Lesser eye: profound visual impairment |
| F490z | Blindness both eyes NOS |
| F491. | Better eye: low vision, Lesser eye: profound visual impairment |
| F4910 | One eye blind, one eye low vision |
| F4911 | Better eye: severe visual impairment, Lesser eye: blind, unspecified |
| F4912 | Better eye: severe visual impairment, Lesser eye: total visual impairment |
| F4913 | Better eye: severe visual impairment, Lesser eye: near total visual impairment |
| F4914 | Better eye: severe visual impairment, Lesser eye: profound visual impairment |
| F4915 | Better eye: moderate visual impairment, Lesser eye: blind, unspecified |
| F4916 | Better eye: moderate visual impairment, Lesser eye: total visual impairment |
| F4917 | Better eye: moderate visual impairment, Lesser eye: near total visual impairment |
| F4918 | Better eye: moderate visual impairment, Lesser eye: profound visual impairment |
| F491z | One eye blind, one eye low vision NOS |
| F492. | Low vision, both eyes |
| F4920 | Low vision, both eyes unspecified |
| F4921 | Better eye: severe visual impairment, Lesser eye: low vision unspecified |
| F4922 | Better eye: severe visual impairment, Lesser eye: severe visual impairment |
| F4923 | Better eye: moderate visual impairment, Lesser eye: low vision unspecified |
| F4924 | Better eye: moderate visual impairment, Lesser eye: severe visual impairment |
| F4925 | Better eye: moderate visual impairment, Lesser eye: moderate visual impairment |
| F492z | Low vision, both eyes NOS |
| F493. | Visual loss, both eyes unqualified |
| F494. | Legal blindness USA |
| F495. | Profound impairment, one eye |
| F4950 | Blindness, one eye, unspecified |
| F4951 | Lesser eye: total visual impairment, Better eye: unspecified |
| F4952 | Lesser eye: total visual impairment, Better eye: near normal vision |
| F4953 | Lesser eye: total visual impairment, Better eye: normal vision |
| F4954 | Lesser eye: near total visual impairment, Better eye: unspecified |
| F4955 | Lesser eye: near total visual impairment, Better eye: near normal vision |
| F4956 | Lesser eye: near total visual impairment, Better eye: normal vision |
| F4957 | Lesser eye: profound visual impairment, Better eye: unspecified |
| F4958 | Lesser eye: profound visual impairment, Better eye: near normal vision |
| F4959 | Lesser eye: profound visual impairment, Better eye: normal vision |
| F495z | Profound impairment one eye NOS |
| F496. | Low vision, one eye |
| F4960 | Low vision, one eye, unspecified |
| F4961 | Lesser eye: severe visual impairment, Better eye: unspecified |
| F4962 | Lesser eye: severe visual impairment, Better eye: near normal vision |
| F4963 | Lesser eye: severe visual impairment, Better eye: normal vision |
| F4964 | Lesser eye: moderate visual impairment, Better eye: unspecified |
| F4965 | Lesser eye: moderate visual impairment, Better eye: near normal vision |
| F4966 | Lesser eye: moderate visual impairment, Better eye: normal vision |
| F496z | Low vision, one eye NOS |
| F49y. | Visual loss, one eye, unqualified |
| F49z. | (Visual loss NOS) or (acquired blindness) |
| Ua1ez | Painful blind eye |
| Ua1f0 | Blindness, monocular |
| X00gP | Partial sight - both eyes |
| X74UF | Provision for visual and hearing impairment |
| X74UG | Provision of guide help for visual and hearing impairment |
| X74UH | Provision of communicator for visual and hearing impairment |
| X75bQ | Weak vision |
| X75bS | Dim vision |
| Xa1Ih | Partial sight |
| XaYfF | Severe binocular visual impairment |
| XaYfG | Severe monocular visual impairment |
| XaYfH | Moderate binocular visual impairment |
| XaYfI | Moderate monocular visual impairment |
| XaYiO | Mild visual impairment |
| XaYiP | Mild binocular visual impairment |
| XaYPJ | Combined visual and hearing impairment |
| XE16L | Visual impairment |
| XE16M | Visual loss NOS |
| XE191 | Blind or low vision - both eyes |
| XE193 | Blind or low vision - one eye only |
| XE195 | Blindness or low vision NOS |
| XM19P | Blind left eye |
| XM19Q | Blind right eye |
| F490. | Blindness - both eyes |
| F4H73 | Cortical blindness |
| FyuJ2 | [X]Other disorders of optic nerve and visual pathways in diseases classified elsewhere |
| FyuL. | [X]Visual disturbances and blindness |
| FyuL0 | [X]Other disorders of binocular vision |
| FyuL1 | [X]Other visual disturbances |
| P356. | (Specified optic disc anomalies) or (optic disc congenital anomalies) |
| X00kS | X-linked sensorineural hearing loss |
| XaaLg | Bilateral congenital sensorineural hearing loss |
| 1C133 | Bilateral deafness |
| XaaLf | Bilateral profound sensorineural hearing loss |
| X00kO | Chronic deafness |
| F5911 | Cochlear hearing loss |
| XaRE1 | Congenital prelingual deafness |
| F5914 | Congenital sensorineural deafness |
| X75qM | Dead ear |
| 1C13. | Deafness (& symptom) |
| XM0Cb | Deafness symptom |
| X00kR | Dominant sensorineural hearing loss |
| XM1QG | Drug ototoxicity - deafness |
| F59.. | Hearing loss (& [deafness]) |
| X00kP | High frequency deafness |
| X00kQ | Low frequency deafness |
| XaOPs | Maternally inherited deafness |
| F592. | Mixed conductive and sensorineural hearing loss |
| F5921 | Mixed conductive and sensorineural hearing loss, bilateral |
| F5920 | Mixed conductive and sensorineural hearing loss, unilateral with unrestricted hearing on the contralateral side |
| XaZuA | Moderate sensorineural hearing loss |
| F5912 | Neural hearing loss |
| XE17O | Ototoxicity - deafness |
| F5915 | Ototoxicity - deafness (& [drug]) |
| 1C132 | Partial deafness |
| X00kU | Perinatal sensorineural hearing loss |
| X00kV | Postnatal acquired sensorineural hearing loss |
| XaZuE | Profound sensorineural hearing loss |
| X00kT | Recessive sensorineural hearing loss |
| XE17N | Sensorineural hearing loss |
| F591. | Sensorineural hearing loss (& [deafness: [high frequency] or [low frequency]) |
| F5916 | Sensorineural hearing loss, bilateral |
| F5917 | Sensorineural hearing loss, unilateral with unrestricted hearing on the contralateral side |
| XaZuB | Severe sensorineural hearing loss |
| 1C131 | Unilateral deafness |
| R0340 | [D]Delayed milestone |
| Ua14s | Global developmental delay |
| X76B7 | Developmental delay |
| Xa40J | Development delay NOS |
| XaX18 | [X]Neurodevelopmental delay |
| XE1hA | O/E - delayed milestones |
| XM1AI | Delayed milestone |
| XE1bg | Developmental delay (& [learning] or [specific]) |
| R03z. | [D]Nutritional, metabolic or developmental symptoms NOS |
| Ub0q0 | Developmental therapy |
| XaLTp | Not yet toilet trained |
| 6665 | Physical handicap problem |
| 6972 | Mobility allowance medical |
| E2F.. | Specific delays in development |
| E2Fy. | Other development delays |
| XaQk5 | History of developmental disorder |
| Y2344 | Developmental Problem |
| ZV402 | [V]Other mental problems |
| 13VC1 | Disability - moderate |
| 13VC2 | Disability - severe |
| 13VC3 | Chronic physical disability |
| 13VC5 | Registered disabled |
| 13VCZ | Disability NOS |
| 9EB4. | DLA 370 Disability living allowance completed |
| E2F5. | Mixed disorder of psychological development |
| E2Fz. | Developmental disorder NOS |
| Eu8.. | Disorder of psychological development |
| Eu83. | [X]Mixed specific developmental disorders |
| Eu8z. | [X]Unspecified disorder of psychological development |
| Ub1S4 | Developmental dysfluency |
| X00TI | Developmental disorder |
| X00TQ | Developmental agnosia |
| XabmM | Early childhood developmental disability |
| XacF5 | Early childhood developmental disability of unknown aetiology |
| XE1aB | [X]Other disorders of psychological development |
| XE1gX | [D]Failure in development NOS |
| XM0zA | Development, failure in [D] |
| XM1AJ | Physiological development failure |
| Y05ac | Toileting problems |
| Y3288 | Development exam result: Unsatisfactory |
| Y6312 | Development, failure in [D] |
| Y6723 | Failure in development NOS [D] |

Regression analyses

*Table S3 – Results of logistic regression model including all co-variables of interest, imputed dataset, odds ratio of* ***developmental disorder*** *by gestational age. Number of observations: 13,172. Number of imputations: 25. Average Relative Variance Increased (RVI) = 0.04; Largest Fraction of Missing Information (FMI) = 0.20. See also Figure 1.*

|  | **Odds ratio** | **95% confidence interval** | | **P value** |
| --- | --- | --- | --- | --- |
| **Gestational age** | | | | |
| <34 weeks | 2.22 | 1.58 | 3.12 | <0.001 |
| 34 - 36 weeks | 1.43 | 1.12 | 1.81 | 0.003 |
| 37 - 38 weeks | 1.18 | 1.03 | 1.34 | 0.02 |
| 39-41 weeks | 1.00 (reference) | | | |
| >41 weeks | 1.39 | 0.91 | 2.13 | 0.1 |
| **Child’s sex** | | | | |
| Male | 2.09 | 1.87 | 2.35 | <0.001 |
| Female | 1.00 (reference) | | | |
| **Small for gestational age (SGA)** | | | | |
| Not SGA | 1.00 (reference) | | | |
| SGA | 1.25 | 1.08 | 1.46 | 0.004 |
| **Maternal age (in years) at child’s delivery** | | | | |
| <21 | 1.03 | 0.83 | 1.29 | 0.8 |
| 21-25 | 1.10 | 0.93 | 1.29 | 0.3 |
| 26-30 | 1.04 | 0.89 | 1.23 | 0.6 |
| 31-35 | 1.00 (reference) | | | |
| >35 | 1.35 | 1.10 | 1.66 | 0.005 |
| **Socio-economic position** | | | | |
| Least deprived | 1.00 (reference) | | | |
| Employed not mat deprived | 1.08 | 0.88 | 1.32 | 0.5 |
| Employed no access to money | 1.33 | 1.08 | 1.64 | 0.007 |
| Benefits but coping | 1.44 | 1.20 | 1.74 | <0.001 |
| Most deprived | 1.52 | 1.24 | 1.87 | <0.001 |
| **Ethnicity** | | | | |
| Asian | 1.00 (reference) | | | |
| White | 1.22 | 1.06 | 1.40 | 0.005 |
| Mixed | 1.15 | 0.89 | 1.48 | 0.3 |
| Black | 0.76 | 0.48 | 1.22 | 0.3 |
| Other | 0.43 | 0.21 | 0.88 | 0.02 |
| **Maternal smoking** | | | | |
| No smoking | 1.00 (reference) | | | |
| Smoking | 1.08 | 0.90 | 1.29 | 0.4 |

*Table S4 – Results of logistic regression model including all co-variables of interest, imputed dataset, odds ratio of* ***Special Educational Need*** *(SEN) provision by gestational age. Number of observations: 11,492. Number of imputations: 25. Average Relative Variance Increased (RVI) = 0.06; Largest Fraction of Missing Information (FMI) = 0.16. See also Figure 2.*

|  | **Odds ratio** | **95% confidence interval** | | **P value** |
| --- | --- | --- | --- | --- |
| **Gestational age** | | | | |
| <34 weeks | 2.10 | 1.52 | 2.90 | <0.001 |
| 34 - 36 weeks | 1.60 | 1.32 | 1.94 | <0.001 |
| 37 - 38 weeks | 1.14 | 1.02 | 1.26 | 0.02 |
| 39-41 weeks | 1.00 (reference) | | | |
| >41 weeks | 1.14 | 0.79 | 1.66 | 0.5 |
| **Child’s sex** | | | | |
| Male | 2.20 | 2.02 | 2.41 | <0.001 |
| Female | 1.00 (reference) | | | |
| **Small for gestational age (SGA)** | | | | |
| Not SGA | 1.00 (reference) | | | |
| SGA | 1.33 | 1.18 | 1.50 | <0.001 |
| **Maternal age (in years) at child’s delivery** | | | | |
| <21 | 1.20 | 1.02 | 1.43 | 0.03 |
| 21-25 | 1.12 | 0.99 | 1.28 | 0.08 |
| 26-30 | 1.08 | 0.95 | 1.23 | 0.2 |
| 31-35 | 1.00 (reference) | | | |
| >35 | 1.12 | 0.95 | 1.33 | 0.2 |
| **Socio-economic position** | | | | |
| Least deprived | 1.00 (reference) | | | |
| Employed not mat deprived | 1.02 | 0.87 | 1.20 | 0.8 |
| Employed no access to money | 1.43 | 1.20 | 1.70 | <0.001 |
| Benefits but coping | 2.01 | 1.73 | 2.33 | <0.001 |
| Most deprived | 2.51 | 2.13 | 2.96 | <0.001 |
| **Ethnicity** | | | | |
| Asian | 1.00 (reference) | | | |
| White | 1.24 | 1.11 | 1.38 | <0.001 |
| Mixed | 1.00 | 0.82 | 1.22 | 1.0 |
| Black | 1.07 | 0.74 | 1.55 | 0.7 |
| Other | 0.51 | 0.31 | 0.83 | 0.006 |
| **Maternal smoking** | | | | |
| No smoking | 1.00 (reference) | | | |
| Smoking | 1.07 | 0.93 | 1.23 | 0.3 |

Missing data and imputation

*Table S5 – Proportion of missing data*

|  | **Developmental disorders,**  **N = 13,172** | | **Special Educational Needs (SEN) provision, N = 11,492** | |
| --- | --- | --- | --- | --- |
|  | N missing | Percentage missing | N missing | Percentage missing |
| Developmental disorder | 0 | 0% |  | |
| SEN provision |  | | 124 | 1.1% |
| Gestational age | 0 | 0% | 0 | 0% |
| Child's sex | 0 | 0% | 0 | 0% |
| Small for Gestational Age | 310 | 2.4% | 280 | 2.4% |
| Maternal age | 0 | 0% | 0 | 0% |
| Ethnicity | 73 | 0.6% | 6 | 0.1% |
| Socio-economic position | 2,329 | 17.7% | 2,044 | 17.8% |
| Maternal smoking | 2,287 | 17.4% | 2,012 | 17.5% |

*Table S6 – Multiple imputation chained equations diagnostics for main analysis.*

|  | **Developmental disorders** | | | **Special Educational Needs (SEN) provision** | | |
| --- | --- | --- | --- | --- | --- | --- |
|  | Observed | Imputed | Completed | Observed | Imputed | Completed |
| No SEN provision |  | | | 72.9% | 79.0% | 72.9% |
| SEN provision |  | | | 27.1% | 21.0% | 27.1% |
| **Small for Gestational Age (SGA)** | | | | | | |
| not SGA | 86.0% | 92.3% | 86.2% | 85.8% | 87.5% | 85.9% |
| SGA | 14.0% | 7.7% | 13.8% | 14.2% | 12.5% | 14.1% |
| **Ethnicity** | | | | | | |
| Asian | 53.2% | 56.2% | 53.2% | 55.0% | 33.3% | 55.0% |
| White | 38.2% | 38.4% | 38.2% | 36.5% | 66.7% | 36.6% |
| Mixed | 5.4% | 2.7% | 5.4% | 5.7% | 0.0% | 5.6% |
| Black | 1.9% | 1.4% | 1.9% | 1.5% | 0.0% | 1.5% |
| Other | 1.4% | 1.4% | 1.4% | 1.4% | 0.0% | 1.4% |
| **Socio-economic position** | | | | | | |
| Least deprived and most educated | 19.3% | 17.0% | 18.9% | 17.6% | 13.80% | 17.0% |
| Employed not materially deprived | 19.9% | 21.3% | 20.2% | 19.7% | 16.90% | 19.2% |
| Employed no access to money | 15.3% | 14.6% | 15.1% | 15.4% | 15.00% | 15.3% |
| Benefits but coping | 29.5% | 30.1% | 29.6% | 30.7% | 34.70% | 31.4% |
| Most deprived | 16.0% | 17.0% | 16.1% | 16.5% | 19.70% | 17.1% |
| **Smoking in pregnancy** | | | | | | |
| no smoking | 83.6% | 83.6% | 83.6% | 83.5% | 83.20% | 83.4% |
| smoking | 16.4% | 16.4% | 16.4% | 16.5% | 16.80% | 16.6% |

Auxiliary variables were considered. Pre-eclampsia, gestational diabetes, parental consanguinity, and maternal parity were associated with the odds of missingness in at least one of the analyses. Therefore, they were included in the imputation as auxiliary variables, in order to maximise the possibility that the missing at random assumption (required to justify the use of multiple imputation) was plausible ^11^. Twenty five imputations were carried out, this exceeded the overall percentage of missing data ^12^. Table S5 shows that the observed and completed values are very similar.

*Table S7 –Results of logistic regression model including all co-variables of interest, odds ratio of* ***developmental disorder****. Complete case analysis.*

|  | **Odds ratio** | **95% confidence interval** | | **P value** |
| --- | --- | --- | --- | --- |
| **Gestational age** | | | | |
| <34 weeks | 2.50 | 1.67 | 3.75 | <0.001 |
| 34 - 36 weeks | 1.37 | 1.02 | 1.82 | 0.03 |
| 37 - 38 weeks | 1.18 | 1.02 | 1.37 | 0.03 |
| 39-41 weeks | 1.00 (reference) | | | |
| >41 weeks | 1.66 | 1.07 | 2.56 | 0.02 |
| **Child’s sex** | | | | |
| Male | 2.16 | 1.90 | 2.46 | <0.001 |
| Female | 1.00 (reference) | | | |
| **Small for gestational age (SGA)** | | | | |
| Not SGA | 1.00 (reference) | | | |
| SGA | 1.25 | 1.06 | 1.49 | 0.01 |
| **Maternal age (in years) at child’s delivery** | | | |  |
| <21 | 0.95 | 0.74 | 1.22 | 0.7 |
| 21-25 | 1.14 | 0.95 | 1.37 | 0.2 |
| 26-30 | 1.03 | 0.86 | 1.24 | 0.8 |
| 31-35 | 1.00 (reference) | | | |
| >35 | 1.38 | 1.10 | 1.74 | 0.006 |
| **Socio-economic position - Five group latent class analysis** | | | | |
| Least deprived | 1.00 (reference) | | | |
| Employed not mat deprived | 1.11 | 0.90 | 1.38 | 0.3 |
| Employed no access to money | 1.41 | 1.13 | 1.76 | 0.002 |
| Benefits but coping | 1.51 | 1.24 | 1.84 | <0.001 |
| Most deprived | 1.56 | 1.25 | 1.94 | <0.001 |
| **Ethnicity** | | | | |
| Asian | 1.00 (reference) | | | |
| White | 1.17 | 1.00 | 1.36 | 0.05 |
| Mixed | 1.01 | 0.75 | 1.35 | 1.0 |
| Black | 0.95 | 0.58 | 1.55 | 0.8 |
| Other | 0.55 | 0.25 | 1.20 | 0.1 |
| **Maternal smoking** | | | | |
| No smoking | 1.00 (reference) | | | |
| Smoking | 1.13 | 0.95 | 1.36 | 0.2 |

*Number of observations: 10,553*

*Table S8 – Results of logistic regression model including all co-variables of interest, odds ratio of* ***Special Educational Need*** *(SEN) provision by gestational age. Complete case analysis.*

|  | **Odds ratio** | **95% confidence interval** | | **P value** |
| --- | --- | --- | --- | --- |
| **Gestational age** | | | | |
| <34 weeks | 2.11 | 1.44 | 3.10 | <0.001 |
| 34 - 36 weeks | 1.66 | 1.32 | 2.10 | <0.001 |
| 37 - 38 weeks | 1.15 | 1.02 | 1.30 | 0.02 |
| 39-41 weeks | 1.00 (reference) | | | |
| >41 weeks | 1.17 | 0.78 | 1.74 | 0.4 |
| **Child’s sex** | | | | |
| Male | 2.26 | 2.05 | 2.50 | <0.001 |
| Female | 1.00 (reference) | | | |
| **Small for gestational age (SGA)** | | | | |
| Not SGA | 1.00 (reference) | | | |
| SGA | 1.36 | 1.19 | 1.56 | <0.001 |
| **Maternal age (in years) at child’s delivery** | | | | |
| <21 | 1.24 | 1.02 | 1.50 | 0.03 |
| 21-25 | 1.21 | 1.04 | 1.40 | 0.01 |
| 26-30 | 1.09 | 0.94 | 1.26 | 0.2 |
| 31-35 | 1.00 (reference) | | | |
| >35 | 1.12 | 0.92 | 1.35 | 0.3 |
| **Socio-economic position - Five group latent class analysis** | | | | |
| Least deprived | 1.00 (reference) | | | |
| Employed not mat deprived | 1.01 | 0.85 | 1.21 | 1.0 |
| Employed no access to money | 1.44 | 1.20 | 1.72 | <0.001 |
| Benefits but coping | 2.05 | 1.75 | 2.39 | <0.001 |
| Most deprived | 2.48 | 2.09 | 2.95 | <0.001 |
| **Ethnicity** | | | | |
| Asian | 1.00 (reference) | | | |
| White | 1.22 | 1.08 | 1.38 | 0.001 |
| Mixed | 0.89 | 0.71 | 1.12 | 0.3 |
| Black | 1.05 | 0.69 | 1.59 | 0.8 |
| Other | 0.51 | 0.28 | 0.93 | 0.03 |
| **Maternal smoking** | | | | |
| No smoking | 1.00 (reference) | | | |
| Smoking | 1.06 | 0.91 | 1.22 | 0.5 |

*Number of observations: 9,084*

Stratified regression analysis

*Table S9 – Results of logistic regression model for* ***developmental disorder*** *stratified by two largest ethnic groups – Complete case analysis*

|  | **Pakistani children N = 4,665** | | | | **White British children N = 3,923** | | | | |
| --- | --- | --- | --- | --- | --- | --- | --- | --- | --- |
|  | Odds ratio | 95% confidence  interval | | P value | | Odds ratio | 95% confidence  interval | | P value |
| Gestational age | | | | | | | | | |
| <34 weeks | 3.60 | 1.99 | 6.52 | <0.0001 | | 1.54 | 0.78 | 3.05 | 0.2 |
| 34 - 36 weeks | 1.47 | 0.93 | 2.32 | 0.1 | | 1.17 | 0.74 | 1.86 | 0.5 |
| 37 - 38 weeks | 1.30 | 1.05 | 1.61 | 0.02 | | 1.13 | 0.88 | 1.45 | 0.3 |
| 39-41 weeks | 1.00 (reference) | | | | | | | | |
| >41 weeks | 1.60 | 0.74 | 3.47 | 0.2 | | 1.55 | 0.83 | 2.89 | 0.2 |
| **Child's sex** | | | | | | | | | |
| Female | 1.00 (reference) | | | | | | | | |
| Male | 1.80 | 1.47 | 2.15 | <0.0001 | | 2.61 | 2.12 | 3.22 | <0.0001 |
| **Small for gestational age (SGA)** | | | | | | | | | |
| Not SGA | 1.00 (reference) | | | | | | | | |
| SGA | 1.29 | 1.02 | 1.62 | 0.03 | | 1 | 0.71 | 1.40 | 0.998 |
| **Maternal age category** | | | | | | | | | |
| <21 | 0.81 | 0.50 | 1.32 | 0.4 | | 0.85 | 0.59 | 1.22 | 0.4 |
| 21-25 | 1.03 | 0.78 | 1.34 | 0.9 | | 1.12 | 0.82 | 1.52 | 0.5 |
| 26-30 | 1.02 | 0.78 | 1.32 | 0.9 | | 0.96 | 0.71 | 1.32 | 0.8 |
| 31-35 weeks | 1.00 (reference) | | | | | | | | |
| >35 | 1.24 | 0.87 | 1.77 | 0.2 | | 1.43 | 0.99 | 2.07 | 0.06 |
| **Socio-economic position** | | | | | | | | | |
| Most educated, least deprived | 1.00 (reference) | | | | | | | | |
| Employed not materially deprived | 1.25 | 0.87 | 1.79 | 0.2 | | 1.25 | 0.89 | 1.76 | 0.2 |
| Employed no access to money | 1.06 | 0.76 | 1.48 | 0.7 | | 1.70 | 1.16 | 2.48 | 0.006 |
| Benefits but coping | 1.16 | 0.89 | 1.51 | 0.3 | | 2.25 | 1.53 | 3.29 | <0.0001 |
| Most deprived | 1.03 | 0.73 | 1.45 | 0.9 | | 2.32 | 1.59 | 3.39 | <0.0001 |
| **Maternal smoking during pregnancy** | | | | | | | | | |
| non-smoking | 1.00 (reference) | | | | | | | | |
| Smoking (any) | 1.04 | 0.63 | 1.71 | 0.9 | | 1.089 | 0.873 | 1.36 | 0.448 |

*Table S10 – Results of logistic regression model for* ***Special Educational Needs*** *provision* *stratified by two largest ethnic groups – Complete case analysis. Mat = materially*

|  | **Pakistani children n = 4,214** | | | | **White British children n = 3,268** | | | |
| --- | --- | --- | --- | --- | --- | --- | --- | --- |
|  | Odds ratio | 95% confidence  interval | | P value | Odds ratio | 95% confidence  interval | | P value |
| Gestational age | | | | | | | | |
| <34 weeks | 3.22 | 1.83 | 5.64 | <0.0001 | 1.35 | 0.71 | 2.57 | 0.4 |
| 34 - 36 weeks | 1.57 | 1.08 | 2.27 | 0.02 | 1.56 | 1.07 | 2.29 | 0.02 |
| 37 - 38 weeks | 1.20 | 1.02 | 1.41 | 0.03 | 1.07 | 0.87 | 1.32 | 0.5 |
| 39-41 weeks | 1.00 (reference) | | | |  | | | |
| >41 weeks | 1.12 | 0.59 | 2.13 | 0.7 | 1.40 | 0.78 | 2.47 | 0.3 |
| Child's sex | | | | | | | | |
| Female | 1.00 (reference) | | | |  | | | |
| Male | 2.21 | 1.92 | 2.54 | <0.0001 | 2.43 | 2.07 | 2.87 | <0.0001 |
| Small for gestational age (SGA) | | | | | | | | |
| Not SGA | 1.00 (reference) | | | |  | | | |
| SGA | 1.35 | 1.13 | 1.61 | 0.001 | 1.19 | 0.91 | 1.57 | 0.2 |
| Maternal age category | | | | | | | | |
| <21 | 1.30 | 0.94 | 1.81 | 0.12 | 1.05 | 0.78 | 1.40 | 0.8 |
| 21-25 | 1.14 | 0.93 | 1.39 | 0.21 | 1.06 | 0.83 | 1.37 | 0.6 |
| 26-30 | 1.06 | 0.87 | 1.29 | 0.57 | 0.97 | 0.76 | 1.26 | 0.8 |
| 31-35 weeks | 1.00 (reference) | | | |  | | | |
| >35 | 0.95 | 0.72 | 1.26 | 0.74 | 1.21 | 0.88 | 1.66 | 0.2 |
| Socio-economic position | | | | | | | | |
| Most educated, least deprived | 1.00 (reference) | | | |  | | | |
| Employed not mat. deprived | 0.96 | 0.71 | 1.30 | 0.8 | 1.11 | 0.84 | 1.47 | 0.5 |
| Employed no access to money | 1.23 | 0.95 | 1.60 | 0.1 | 1.64 | 1.20 | 2.24 | 0.002 |
| Benefits but coping | 1.77 | 1.43 | 2.18 | 0.001 | 2.69 | 1.96 | 3.69 | <0.0001 |
| Most deprived | 1.93 | 1.49 | 2.49 | 0.001 | 2.98 | 2.19 | 4.05 | <0.0001 |
| Maternal smoking during pregnancy | | | | | | | | |
| non-smoking | 1.00 (reference) | | | |  | | | |
| Smoking (any) | 0.96 | 0.65 | 1.41 | 0.8 | 1.04 | 0.87 | 1.25 | 0.7 |

*Table S11 – Multiple imputation chained equations diagnostics for analysis stratified by ethnicity.*

| **Developmental disorders** | | | | | | |
| --- | --- | --- | --- | --- | --- | --- |
|  | **Pakistani children** | | | **White British children** | | |
|  | **observed** | **imputed** | **completed** | **observed** | **imputed** | **completed** |
| **Socio-economic position** | | | | | | |
| n | 4812 | 976 | 5788 | 4029 | 595 | 4624 |
| Least deprived and most educated | 0.187 | 0.164 | 0.183 | 0.167 | 0.123 | 0.161 |
| Employed not materially deprived | 0.096 | 0.101 | 0.097 | 0.331 | 0.259 | 0.322 |
| Employed no access to money | 0.146 | 0.132 | 0.143 | 0.156 | 0.160 | 0.157 |
| Benefits but coping | 0.437 | 0.473 | 0.443 | 0.159 | 0.173 | 0.161 |
| Most deprived | 0.134 | 0.129 | 0.133 | 0.187 | 0.286 | 0.200 |
| **Smoking during pregnancy** | | | | | | |
| n | 4814 | 974 | 5788 | 4039 | 585 | 4624 |
| no smoking | 0.965 | 0.965 | 0.965 | 0.675 | 0.583 | 0.663 |
| smoked | 0.035 | 0.035 | 0.035 | 0.325 | 0.417 | 0.337 |
| **Small for gestational age (SGA)** | | | | | | |
| n | 5641 | 147 | 5788 | 4515 | 109 | 4624 |
| not SGA | 0.826 | 0.837 | 0.826 | 0.906 | 0.936 | 0.907 |
| SGA | 0.174 | 0.163 | 0.174 | 0.094 | 0.064 | 0.093 |
| **Special educational needs (SEN) provision** | | | | | | |
|  | **Pakistani children** | | | **White British children** | | |
| **SEN status** | | | | | | |
| n | 5250 | 49 | 5299 | 3876 | 39 | 3915 |
| No SEN | 0.725 | 0.837 | 0.726 | 0.709 | 0.744 | 0.710 |
| SEN | 0.275 | 0.163 | 0.274 | 0.291 | 0.256 | 0.290 |
| **Socio-economic position** | | | | | | |
| n | 4286 | 913 | 5299 | 3398 | 517 | 3915 |
| Least deprived and most educated | 0.174 | 0.165 | 0.173 | 0.154 | 0.089 | 0.145 |
| Employed not materially deprived | 0.097 | 0.094 | 0.097 | 0.332 | 0.313 | 0.330 |
| Employed no access to money | 0.145 | 0.138 | 0.144 | 0.159 | 0.166 | 0.160 |
| Benefits but coping | 0.444 | 0.473 | 0.449 | 0.161 | 0.176 | 0.163 |
| Most deprived | 0.139 | 0.129 | 0.138 | 0.195 | 0.255 | 0.203 |
| **Smoking during pregnancy** | | | | | | |
| n | 4385 | 914 | 5299 | 3406 | 509 | 3915 |
| no smoking | 0.968 | 0.968 | 0.968 | 0.661 | 0.593 | 0.652 |
| smoked | 0.032 | 0.032 | 0.032 | 0.339 | 0.407 | 0.348 |
| **Small for gestational age (SGA)** | | | | | | |
| n | 5167 | 132 | 5299 | 3815 | 100 | 3915 |
| not SGA | 0.824 | 0.826 | 0.824 | 0.907 | 0.900 | 0.907 |
| SGA | 0.176 | 0.174 | 0.176 | 0.093 | 0.100 | 0.093 |

Incidence rates of individual disorders

*Table S12 – Incidence rate of each disorder/ group of disorders, sorted by incidence rate*

| **Developmental disorder** | **person-years** | **Cases** | **Incidence rate**  **(/ 1000 person years)** | **95% confidence**  **interval** | |
| --- | --- | --- | --- | --- | --- |
| Speech, language, and communication disorders | 172,328.3 | 606 | 3.52 | 3.25 | 3.81 |
| Autism spectrum disorder | 176,407.5 | 377 | 2.14 | 1.93 | 2.36 |
| Social, emotional, and behavioral problems | 176,426.5 | 359 | 2.03 | 1.83 | 2.26 |
| Education problems | 176,004.7 | 340 | 1.93 | 1.74 | 2.15 |
| Developmental delay | 175,405.0 | 277 | 1.58 | 1.40 | 1.78 |
| Sleep apnoea | 176,433.6 | 222 | 1.26 | 1.1 | 1.44 |
| Learning disability | 177,095.3 | 209 | 1.18 | 1.03 | 1.35 |
| Attention, impulsivity and hyperactivity including ADHD | 177,638.9 | 157 | 0.88 | 0.76 | 1.03 |
| Motor function problems | 177,308.2 | 94 | 0.53 | 0.43 | 0.65 |
| Visual impairment | 177,457.7 | 88 | 0.50 | 0.4 | 0.61 |
| General developmental disorder | 177,393.0 | 87 | 0.49 | 0.40 | 0.61 |
| Hearing impairment | 177,434.7 | 76 | 0.43 | 0.34 | 0.54 |
| Feeding problems | 177,641.3 | 51 | 0.29 | 0.22 | 0.38 |
| Cerebral palsy | 177,739.8 | 44 | 0.25 | 0.18 | 0.33 |

*Table S13 - Incidence rate per 1000 person years of developmental disorders by ethnicity*

|  | **Pakistani children** | | | **White British children** | | |
| --- | --- | --- | --- | --- | --- | --- |
| **Developmental disorder** | **Rate / 1000 person years** | **lower 95%CI** | **upper 95% CI** | **Rate / 1000 person years** | **lower 95%CI** | **upper 95% CI** |
| Cerebral palsy | 0.36 | 0.25 | 0.52 | 0.19 | 0.11 | 0.34 |
| Motor function problems | 0.62 | 0.47 | 0.82 | 0.59 | 0.43 | 0.82 |
| Learning disability | 1.62 | 1.36 | 1.93 | 0.72 | 0.54 | 0.96 |
| Education problems | 2.32 | 2.01 | 2.69 | 1.66 | 1.37 | 2.01 |
| Speech/language/communication | 3.32 | 2.93 | 3.75 | 3.95 | 3.48 | 4.48 |
| ADHD | 0.27 | 0.18 | 0.41 | 1.75 | 1.45 | 2.11 |
| ASD | 1.27 | 1.04 | 1.55 | 3.53 | 3.10 | 4.03 |
| Feeding problems | 0.37 | 0.26 | 0.54 | 0.16 | 0.09 | 0.30 |
| Sleep apnoea | 1.19 | 0.97 | 1.46 | 1.22 | 0.98 | 1.53 |
| Social/Emotional/Behavioural problems | 1.40 | 1.16 | 1.69 | 3.16 | 2.75 | 3.63 |
| Hearing impairment | 0.59 | 0.45 | 0.79 | 0.27 | 0.17 | 0.44 |
| Visual impairment | 0.59 | 0.44 | 0.79 | 0.40 | 0.27 | 0.59 |
| Developmental delay | 1.95 | 1.66 | 2.29 | 1.44 | 1.17 | 1.77 |
| General | 0.62 | 0.47 | 0.82 | 0.40 | 0.27 | 0.59 |

Sensitivity analysis: Restricted case ascertainment strategy

Excluding codes relating to sleep apnoea results in a cumulative incidence of any developmental disorder of 10.1% (9.54 to 10.6%). When restricting further to only diagnostic codes corresponding to DSM-IV or ICD-10 developmental disorders (i.e. excluding codes related to developmental ‘problems’, signs, or symptoms) the cumulative incidence was 8.9% (8.4 to 9.4%), see table S13.

Table S14- Cumulative incidence of developmental disorder using unrestricted and restricted case ascertainment strategies

|  | **Number of incident cases** | **Cumulative incidence**  **(95% confidence intervals)** |
| --- | --- | --- |
| **Unrestricted case ascertainment strategy (main results)** | | |
| Primary care | 1,355 | 10.3% (9.8 to 10.8%) |
| Secondary care | 524 | 4.0% (3.7 to 4.3%) |
| Combined | 1,497 | **11.4%** (10.8 to 11.9%) |
| **Partially restricted case ascertainment strategy** – excluding sleep apnoea codes | | |
| Primary care | 1,224 | 9.3% (8.8 to 9.8%) |
| Secondary care | 399 | 3.0% (2.7 to 3.3%) |
| Combined | 1,330 | **10.1%** (9.5 to 10.6%) |
| **Fully restricted case ascertainment strategy** – excluding sleep apnoea codes and codes relating to developmental ‘problems’, signs, or symptoms | | |
| Primary care | 1,110 | 8.4% (8.0 to 8.9%) |
| Secondary care | 321 | 2.4% (2.2 to 2.7%) |
| Combined | 1,117 | **8.9%** (8.4 to 9.4%) |

The imputed multivariable regression model was repeated but using the fully restricted case ascertainment strategy. The effect sizes are similar to the main model, although slightly larger see table S14, e.g. odds ratio of developmental disorder <34 weeks in restricted model 2.41 (95% confidence intervals 1.67 to 3.47) vs. 2.22 (95% CI 1.58 to 3.12) in main model.

Table S15. Odds ratio of developmental disorder according to gestational age. Fully adjusted model. N = 13,172. Number of imputations: 25; Average Relative Variance Increased (RVI): 0.049; Largest Fraction of Missing Information (FMI):0.18

|  | **Odds ratio** | **95% confidence interval** | | **P value** |
| --- | --- | --- | --- | --- |
| **Gestational age** | | | | |
| <34 weeks | 2.41 | 1.67 | 3.47 | <0.001 |
| 34 - 36 weeks | 1.46 | 1.12 | 1.90 | 0.005 |
| 37 - 38 weeks | 1.26 | 1.09 | 1.46 | 0.001 |
| 39-41 weeks | 1 (reference) | |  |  |
| >41 weeks | 1.54 | 0.97 | 2.43 | 0.07 |
| **Child's sex** | | | | |
| Female | 1 (reference) | |  |  |
| Male | 2.27 | 1.99 | 2.58 | <0.001 |
| **Small for gestational age (SGA)** | | | | |
| not SGA | 1 (reference) | |  |  |
| SGA | 1.29 | 1.09 | 1.53 | 0.003 |
| **Maternal age at delivery (years)** | | | | |
| <21 | 1.03 | 0.81 | 1.32 | 0.8 |
| 21-25 | 1.08 | 0.90 | 1.30 | 0.4 |
| 26-30 | 1.09 | 0.91 | 1.30 | 0.4 |
| 31-35 | 1 (reference) | |  |  |
| >35 | 1.41 | 1.13 | 1.78 | 0.003 |
| **Socio-economic position** | | | | |
| Most educated, least deprived | 1 (reference) | |  |  |
| Employed not materially deprived | 21.10 | 0.88 | 1.41 | 0.4 |
| Employed no access to money | 1.31 | 1.03 | 1.66 | 0.03 |
| Benefits but coping | 1.45 | 1.18 | 1.78 | <0.001 |
| Most deprived | 1.48 | 1.17 | 1.87 | 0.001 |
| **Maternal smoking in pregnancy** | | | | |
| Non-smoking | |  |  |  |
| Smoking (any) | 1.02 | 0.84 | 1.25 | 0.8 |
| **Ethnicity** | | | | |
| White | 1.22 | 1.05 | 1.42 | 0.01 |
| Mixed | 1.22 | 0.93 | 1.61 | 0.1 |
| Asian | 1 (reference) | |  |  |
| Black | 0.73 | 0.43 | 1.25 | 0.3 |
| Other | 0.35 | 0.14 | 0.85 | 0.02 |

Sensitivity analysis: Time to event analysis

*Table S16. Kaplan-Meier Estimates of Time to First Developmental Disorder Diagnosis by Gestational Age Group*

| **Time (months)** | **Number of children at risk** | **number of children diagnosed with dev. disorder** | **Survivor function** | **Std. error** | **95% conf. interval** | |
| --- | --- | --- | --- | --- | --- | --- |
| <34 weeks | | | | | | |
| 24 | 186 | 16 | 0.922 | 0.019 | 0.876 | 0.952 |
| 48 | 172 | 14 | 0.852 | 0.025 | 0.796 | 0.894 |
| 72 | 165 | 7 | 0.817 | 0.027 | 0.757 | 0.864 |
| 96 | 162 | 3 | 0.802 | 0.028 | 0.741 | 0.851 |
| 120 | 158 | 3 | 0.788 | 0.029 | 0.724 | 0.838 |
| 144 | 155 | 3 | 0.772 | 0.030 | 0.708 | 0.824 |
| 34 - 36 weeks | | | | | | |
| 24 | 579 | 18 | 0.970 | 0.007 | 0.953 | 0.981 |
| 48 | 549 | 30 | 0.920 | 0.011 | 0.895 | 0.939 |
| 72 | 531 | 17 | 0.891 | 0.013 | 0.863 | 0.913 |
| 96 | 521 | 8 | 0.877 | 0.013 | 0.848 | 0.901 |
| 120 | 513 | 5 | 0.869 | 0.014 | 0.839 | 0.894 |
| 144 | 498 | 11 | 0.850 | 0.015 | 0.819 | 0.877 |
| 37 - 38 weeks | | | | | | |
| 24 | 2857 | 59 | 0.980 | 0.003 | 0.974 | 0.984 |
| 48 | 2740 | 106 | 0.943 | 0.004 | 0.934 | 0.951 |
| 72 | 2643 | 87 | 0.913 | 0.005 | 0.902 | 0.923 |
| 96 | 2591 | 46 | 0.897 | 0.006 | 0.886 | 0.908 |
| 120 | 2553 | 32 | 0.886 | 0.006 | 0.874 | 0.897 |
| 144 | 2489 | 31 | 0.875 | 0.006 | 0.863 | 0.887 |
| 39 - 41 weeks | | | | | | |
| 24 | 8953 | 146 | 0.984 | 0.001 | 0.981 | 0.986 |
| 48 | 8634 | 306 | 0.950 | 0.002 | 0.946 | 0.955 |
| 72 | 8378 | 224 | 0.926 | 0.003 | 0.920 | 0.931 |
| 96 | 8249 | 114 | 0.913 | 0.003 | 0.907 | 0.919 |
| 120 | 8125 | 92 | 0.903 | 0.003 | 0.897 | 0.909 |
| 144 | 7864 | 95 | 0.892 | 0.003 | 0.886 | 0.898 |
| >41 weeks | | | | | | |
| 24 | 173 | 4 | 0.977 | 0.011 | 0.941 | 0.991 |
| 48 | 167 | 6 | 0.943 | 0.017 | 0.897 | 0.969 |
| 72 | 160 | 7 | 0.904 | 0.022 | 0.849 | 0.939 |
| 96 | 158 | 2 | 0.892 | 0.023 | 0.836 | 0.930 |
| 120 | 157 | 1 | 0.886 | 0.024 | 0.830 | 0.925 |
| 144 | 148 | 6 | 0.852 | 0.027 | 0.791 | 0.897 |

*Table S17. Kaplan-Meier Estimates of Time to First Special Educational Needs (SEN) by Gestational Age Group*

| **Time (months)** | **Number of children at risk** | **number of children ‘diagnosed’ with SEN** | **Survivor function** | **Std.error** | **95% conf. interval** | |
| --- | --- | --- | --- | --- | --- | --- |
| <34 weeks | | | | | | |
| 72 | 150 | 25 | 0.855 | 0.027 | 0.793 | 0.899 |
| 96 | 120 | 29 | 0.686 | 0.035 | 0.611 | 0.750 |
| 120 | 111 | 7 | 0.645 | 0.037 | 0.569 | 0.712 |
| 144 | 104 | 7 | 0.604 | 0.037 | 0.527 | 0.673 |
| 34 - 36 weeks | | | | | | |
| 72 | 492 | 40 | 0.924 | 0.012 | 0.898 | 0.944 |
| 96 | 402 | 88 | 0.757 | 0.019 | 0.7179 | 0.791 |
| 120 | 358 | 37 | 0.686 | 0.020 | 0.645 | 0.724 |
| 144 | 335 | 21 | 0.646 | 0.021 | 0.603 | 0.685 |
| 37 - 38 weeks | | | | | | |
| 72 | 2455 | 172 | 0.934 | 0.005 | 0.924 | 0.943 |
| 96 | 2124 | 318 | 0.812 | 0.008 | 0.797 | 0.827 |
| 120 | 1966 | 146 | 0.756 | 0.008 | 0.739 | 0.772 |
| 144 | 1890 | 71 | 0.729 | 0.009 | 0.711 | 0.745 |
| 39 - 41 weeks | | | | | | |
| 72 | 7632 | 419 | 0.948 | 0.003 | 0.943 | 0.952 |
| 96 | 6653 | 962 | 0.828 | 0.004 | 0.819 | 0.836 |
| 120 | 6167 | 429 | 0.774 | 0.005 | 0.765 | 0.783 |
| 144 | 5925 | 212 | 0.747 | 0.005 | 0.738 | 0.757 |
| >41 weeks | | | | | | |
| 72 | 142 | 10 | 0.934 | 0.020 | 0.880 | 0.964 |
| 96 | 123 | 19 | 0.808 | 0.032 | 0.736 | 0.862 |
| 120 | 115 | 8 | 0.755 | 0.035 | 0.678 | 0.816 |
| 144 | 108 | 5 | 0.722 | 0.037 | 0.643 | 0.786 |

*Table S18 - Cox Proportional Hazards Model for Time to First diagnosis of Developmental disorder (Complete case analysis)*

|  | Hazard ratio | Std. err. | z | P>z | 95% conf.  interval | |
| --- | --- | --- | --- | --- | --- | --- |
| Gestational age | | | | | | |
| <34 weeks | 2.323 | 0.401 | 4.88 | <0.0001 | 1.656 | 3.259 |
| 34 - 36 weeks | 1.332 | 0.171 | 2.24 | 0.03 | 1.036 | 1.712 |
| 37 - 38 weeks | 1.158 | 0.078 | 2.18 | 0.03 | 1.015 | 1.32 |
| 39 – 41 weeks | 1 (ref) | | | | | |
| >41 weeks | 1.508 | 0.295 | 2.1 | 0.04 | 1.027 | 2.213 |
| Baby’s sex | | | | | | |
| Female | 1 (ref) | | | | | |
| Male | 1.979 | 0.115 | 11.71 | <0.0001 | 1.766 | 2.219 |
| Small for Gestational age | | | | | | |
| Not SGA | 1 (ref) | | | | | |
| SGA | 1.233 | 0.095 | 2.72 | 0.006 | 1.06 | 1.433 |
| Maternal age | | | | | | |
| <20 yrs | 0.874 | 0.112 | -1.05 | 0.295 | 0.679 | 1.124 |
| 20-24yrs | 1.065 | 0.087 | 0.76 | 0.444 | 0.907 | 1.25 |
| 25-29yrs | 1.004 | 0.079 | 0.05 | 0.959 | 0.861 | 1.171 |
| 30 – 34 years | 1 (ref) | | | | | |
| 35-39yrs | 1.188 | 0.121 | 1.7 | 0.09 | 0.974 | 1.45 |
| >40 yrs | 1.433 | 0.252 | 2.05 | 0.04 | 1.016 | 2.022 |
| Socio-economic status | | | | | | |
| Least deprived | 1 (ref) | | | | | |
| Employed not mat dep | 1.065 | 0.105 | 0.64 | 0.521 | 0.878 | 1.292 |
| Emp. no access to money | 1.344 | 0.135 | 2.94 | 0.003 | 1.104 | 1.637 |
| Benefits but coping | 1.487 | 0.134 | 4.39 | <0.0001 | 1.245 | 1.775 |
| Most deprived | 1.552 | 0.154 | 4.43 | <0.0001 | 1.278 | 1.885 |
| Ethnicity | | | | | | |
| Asian | 1 (ref) | | | | | |
| White | 1.284 | 0.088 | 3.64 | <0.0001 | 1.122 | 1.469 |
| Mixed | 1.071 | 0.141 | 0.52 | 0.602 | 0.827 | 1.387 |
| Black | 0.891 | 0.209 | -0.49 | 0.621 | 0.563 | 1.41 |
| Other | 0.525 | 0.2 | -1.69 | 0.091 | 0.249 | 1.109 |
| Smoking | | | | | | |
| Non-smoker | 1 (ref) | | | | | |
| Smoker | 1.15 | 0.091 | 1.78 | 0.075 | 0.986 | 1.342 |

*Number of obs = 10,532*

Gestational age, ethnicity and smoking likely violate the proportional hazards assumption, and therefore were included as time variable covariates using an interaction with log (time), see Table S19.

*Table S19 - Cox Proportional Hazards Model for Time to First diagnosis of Developmental disorder including time-varying covariates using an interaction with log(time) (Complete case analysis)*

|  | Haz. ratio | Std. err. | z | P>z | 95% conf.  interval | |
| --- | --- | --- | --- | --- | --- | --- |
| Gestational age | | | | | | |
| <34 weeks | 9.11 | 4.278 | 4.7 | <0.0001 | 3.629 | 22.86 |
| 34 - 36 weeks | 3.36 | 1.098 | 3.7 | <0.0001 | 1.767 | 6.371 |
| 37 - 38 weeks | 1.84 | 0.308 | 3.65 | <0.0001 | 1.327 | 2.5561 |
| 39 – 41 weeks | 1 (ref) | | | | | |
| >41 weeks | 0.93 | 0.236 | -0.28 | 0.782 | 0.568 | 1.532 |
| Baby’s sex | | | | | | |
| Male | 1.98 | 0.116 | 11.75 | <0.0001 | 1.770 | 2.225 |
| Female | 1 (ref) | | | | | |
| SGA | | | | | | |
| Not SGA | 1 (ref) | | | | | |
| SGA | 1.228 | 0.094 | 2.68 | 0.007 | 1.057 | 1.428 |
| Maternal age | | | | | | |
| <20 yrs | 0.873 | 0.112 | -1.05 | 0.292 | 0.679 | 1.124 |
| 20-24yrs | 1.068 | 0.088 | 0.80 | 0.422 | 0.909 | 1.254 |
| 25-29yrs | 1.008 | 0.079 | 0.10 | 0.924 | 0.864 | 1.175 |
| 30-34 years | 1 (ref) | | | | | |
| 35-39yrs | 1.190 | 0.121 | 1.72 | 0.086 | 0.976 | 1.4527 |
| >40 yrs | 1.423 | 0.250 | 2.01 | 0.044 | 1.009 | 2.008 |
| Socio-economic position | | | | | | |
| Least deprived | 1 (ref) | | | | | |
| Employed not mat dep | 1.065 | 0.105 | 0.63 | 0.525 | 0.878 | 1.291 |
| Emp. no access to money | 1.346 | 0.135 | 2.95 | 0.003 | 1.105 | 1.638 |
| Benefits but coping | 1.489 | 0.135 | 4.41 | <0.0001 | 1.247 | 1.777 |
| Most deprived | 1.558 | 0.155 | 4.47 | <0.0001 | 1.283 | 1.892 |
| Ethnicity | | | | | | |
| Asian | 1 (ref) | | | | | |
| White | 0.175 | 0.053 | -5.8 | <0.0001 | 0.097 | 0.315 |
| Mixed | 0.409 | 0.079 | -4.64 | <0.0001 | 0.280 | 0.596 |
| Black | 2.224 | 0.589 | 3.02 | 0.003 | 1.323 | 3.737 |
| Other | 3.068 | 1.356 | 2.54 | 0.011 | 1.290 | 7.296 |
| Smoking | | | | | | |
| Non-smoker | 1 (ref) | | | | | |
| Smoker | 0.309 | 0.128 | -2.83 | 0.005 | 0.137 | 0.695 |
| time-varying covariates | | | | | | |
| Gestational age | 1.127 | 0.044 | 3.04 | 0.002 | 1.043 | 1.217 |
| Ethnicity | 0.776 | 0.028 | -6.92 | 0 | 0.721 | 0.833 |
| Smoking | 1.381 | 0.136 | 3.27 | 0.001 | 1.137 | 1.6750 |

*Table S20 – Cox Proportional Hazards Model for Time to First Special Educational Needs (SEN) (complete case)*

|  | Haz.ratio | Std. err. | z | P>z | 95% conf.  interval | |
| --- | --- | --- | --- | --- | --- | --- |
| Gestational age | | | | | | |
| <34 weeks | 1.754 | 0.255 | 3.87 | <0.0001 | 1.319 | 2.331 |
| 34 - 36 weeks | 1.47 | 0.133 | 4.26 | <0.0001 | 1.232 | 1.755 |
| 37 - 38 weeks | 1.11 | 0.054 | 2.14 | 0.032 | 1.009 | 1.222 |
| 39-41 weeks | 1 (ref) | | | | | |
| >41 weeks | 1.14 | 0.19 | 0.79 | 0.43 | 0.823 | 1.58 |
| Baby’s sex | | | | | | |
| Female | 1 (ref) | | | | | |
| Male | 1.96 | 0.082 | 16.18 | <0.0001 | 1.807 | 2.127 |
| Small for Gestational age | | | | | | |
| Not SGA | 1 (ref) | | | | | |
| SGA | 1.286 | 0.07 | 4.62 | <0.0001 | 1.156 | 1.43 |
| Maternal age | | | | | | |
| <20 yrs | 1.113 | 0.099 | 1.2 | 0.23 | 0.934 | 1.326 |
| 20-24yrs | 1.179 | 0.071 | 2.74 | 0.006 | 1.048 | 1.326 |
| 25-29yrs | 1.104 | 0.064 | 1.72 | 0.086 | 0.986 | 1.236 |
| 30 – 34 years | 1 (ref) | | | | | |
| 35-39yrs | 1.112 | 0.086 | 1.37 | 0.17 | 0.955 | 1.294 |
| >40 yrs | 1.311 | 0.176 | 2.02 | 0.044 | 1.007 | 1.706 |
| Socio-economic status | | | | | | |
| Least deprived | 1 (ref) | | | | | |
| Employed not mat dep | 1.022 | 0.08 | 0.28 | 0.782 | 0.877 | 1.191 |
| Emp.no access to money | 1.369 | 0.107 | 4.01 | 0 | 1.174 | 1.596 |
| Benefits but coping | 1.854 | 0.127 | 9.02 | 0 | 1.621 | 2.12 |
| Most deprived | 2.122 | 0.156 | 10.21 | 0 | 1.837 | 2.452 |
| Ethnicity | | | | | | |
| Asian | 1 (ref) | | | | | |
| White | 1.189 | 0.061 | 3.41 | 0.001 | 1.077 | 1.314 |
| Mixed | 0.909 | 0.088 | -0.99 | 0.32 | 0.752 | 1.098 |
| Black | 1 | 0.178 | 0 | 0.998 | 0.706 | 1.416 |
| Other | 0.508 | 0.142 | -2.42 | 0.015 | 0.294 | 0.879 |
| Smoking | | | | | | |
| Non-smoker | 1 (ref) | | | | | |
| Smoker | 1.033 | 0.061 | 0.55 | 0.581 | 0.92 | 1.16 |

*No. of obs = 9,182*

In this regression, sex, socio-economic status and ethnicity violate the proportional hazards assumptions. Therefore, they were included as time varying constants (see Table S21)

*Table S21 - Cox Proportional Hazards Model for Time to SEN including time-varying covariates using an interaction with log(time)*

|  | Haz. ratio | Std. err. | z | p | | 95% conf.  interval | |
| --- | --- | --- | --- | --- | --- | --- | --- |
| Gestational age | | | | | | | |
| <34 weeks | 1.741 | 0.252 | 3.82 | <0.0001 | 1.310 | | 2.314 |
| 34 - 36 weeks | 1.474 | 0.133 | 4.29 | <0.0001 | 1.234 | | 1.759 |
| 37 - 38 weeks | 1.108 | 0.054 | 2.11 | 0.035 | 1.007 | | 1.219 |
| 39-41 weeks | 1 (ref) | | | | | | |
| >41 weeks | 1.141 | 0.189 | 0.79 | 0.427 | 0.823 | | 1.581 |
| Child’s sex | | | | | | | |
| Male | Ref (1) | | | | | | |
| Female | 0.167 | 0.120 | -2.49 | 0.013 | 0.041 | | 0.683 |
| Small for Gestational age | | | | | | | |
| Not SGA | 1 (ref) | | | | | | |
| SGA | 1.282 | 0.069 | 4.58 | <0.0001 | 1.153 | | 1.426 |
| Maternal age | | | | | | | |
| <20 yrs | 1.106 | 0.098 | 1.13 | 0.258 | 0.928 | | 1.318 |
| 20-24yrs | 1.176 | 0.070 | 2.70 | 0.007 | 1.045 | | 1.322 |
| 25-29yrs | 1.101 | 0.063 | 1.68 | 0.093 | 0.984 | | 1.233 |
| 30-24 years | 1 (ref) | | | | | | |
| 35-39yrs | 1.110 | 0.085 | 1.36 | 0.175 | 0.954 | | 1.292 |
| >40 yrs | 1.306 | 0.175 | 1.99 | 0.046 | 1.004 | | 1.700 |
| Socio-economic position | | | | | | | |
| Least deprived | 1 (ref) | | | | | | |
| Employed not mat dep | 0.449 | 0.121 | -2.96 | 0.003 | 0.264 | | 0.764 |
| Employed no access to money | 0.263 | 0.139 | -2.52 | 0.012 | 0.093 | | 0.743 |
| Benefits but coping | 0.156 | 0.123 | -2.35 | 0.019 | 0.033 | | 0.735 |
| Most dep | 0.078 | 0.082 | -2.42 | 0.016 | 0.009 | | 0.617 |
| Ethnicity | | | | | | | |
| Asian | 1 (ref) | | | | | | |
| White | 0.004 | 0.003 | -7.56 | <0.0001 | 0.001 | | 0.019 |
| Mixed | 0.058 | 0.021 | -7.84 | <0.0001 | 0.028 | | 0.1183 |
| Black | 15.387 | 5.968 | 7.05 | <0.0001 | 7.194 | | 32.909 |
| Other | 118.12 | 87.32 | 6.46 | <0.0001 | 27.74 | | 503.014 |
| Smoking | | | | | | | |
| Non-smoker | 1 (ref) | | | | | | |
| Smoker | 1.032 | 0.060 | 0.54 | 0.590 | 0.919 | | 1.158 |
| time-varying covariates | | | | | | | |
| Child’s sex | 1.279 | 0.203 | 1.55 | 0.121 | 0.936 | | 1.748 |
| Socio-economic position | 1.201 | 0.070 | 3.14 | 0.002 | 1.071 | | 1.348 |
| Ethnicity | 0.541 | 0.042 | -7.84 | <0.0001 | 0.464 | | 0.630 |
